# Radical-Mediated Trifunctionalization Reactions

**DOI:** 10.3390/molecules29153620

**Published:** 2024-07-31

**Authors:** Qiang Zhang, Xiaoming Ma, Sanjun Zhi, Wei Zhang

**Affiliations:** 1 School of Chemistry and Life Sciences, Suzhou University of Science and Technology, 99 Xuefu Road, Suzhou 215009, China; 2 School of Pharmacy, Changzhou University, 1 Gehu Road, Changzhou 213164, China; mxm.wuxi@cczu.edu.cn; 3 Jiangsu Key Laboratory for the Chemistry of Low-Dimensional Materials, Huaiyin Normal University, 111 Changjiang West Road, Huaian 223300, China; 4 Department of Chemistry, University of Massachusetts Boston, 100 Morrissey Boulevard, Boston, MA 02125, USA

**Keywords:** radical, trifunctionalization, alkene, alkyne, addition, cyclization, atom transfer, group transfer

## Abstract

Synthetic radicals have intrinsic power for cascading and multifunctional reactions to construct diverse molecular scaffolds. In the previous review series, we covered 1,2-difunctionalizations, remote 1,3-, 1,4-, 1,5-, 1,6-, and 1,7-difunctionalizations, addition followed by cyclization reactions, and cycloaddition-initiated difunctionalizations. Presented in this paper are radical addition-initiated trifunctionalization reactions of alkenes, alkynes, and their derivatives. After the initial radical addition, there are different pathways, such as group or hydrogen atom transfer, cyclization, and radical coupling, to complete the second and third functionalizations.

## 1. Introduction

**Scheme 1:**
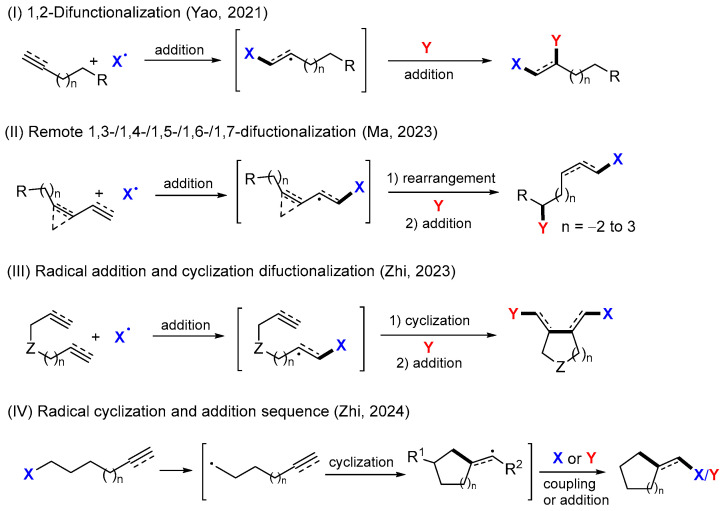
Radical difunctionalization reactions [32,33,34,35].

Highly reactive synthetic radicals are feasible in making carbon–carbon and carbon–heteroatom bonds via a broad scope of transformations, including addition, cyclization, coupling, rearrangement, fragmentation, atom or group transfer, and single electron oxidation/reduction reactions [1,2]. Cascade radical transformations have intrinsic power in the construction of diverse molecular scaffolds and for incorporating multiple functional groups into the products. In addition to traditional methods for conducting radical reactions [3,4,5,6,7,8,9], recent developments in photoredox reactions [10,11,12,13], electrochemical reactions [14,15], and mechanoredox reactions [16] have paved new ways for synthetic radicals. From reaction efficiency, product diversity, and green chemistry considerations, cascade reactions and difunctionalization have a high pot-, atom-, and step-economy [17,18,19]. There are a number of reviews on radical difunctionalization reactions [20,21,22,23,24,25,26,27,28,29,30,31], including our recent articles on 1,2-difunctionalization of alkenes and alkynes (Scheme 1I) [32], remote 1,3-, 1,4-, 1,5-, 1,6-, and 1,7-difunctionalization of alkenes and alkynes (Scheme 1II) [33], addition/cyclization sequence of dienes, enynes, and related compounds (Scheme 1III) [34], and cyclization-initiated sequence for making cyclic compounds (Scheme 1IV) [35]. This paper covers radical trifunctionalization reactions reported in the last decade, while most papers were published in the last five years.

**Scheme 2:**
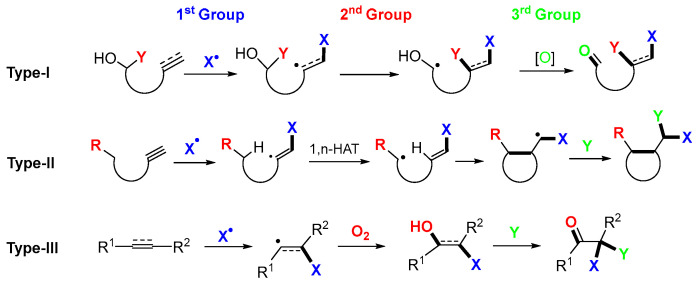
Three representative radical trifunctionalization reactions.

The radical trifunctionalization presented in this paper cannot be summarized by a single reaction scheme like that for the difunctional reactions shown in Scheme 1. Three representative radical trifunctionalization reactions are shown in Scheme 2. Alkenols or alkynols are employed as substrates in the Type-I reaction. The initial radical addition introduces X as the first group, followed by the atom or group transfer of Y as the second group. Oxidation of hydroxyl-carbon to carbonyl introduces the third group. The Type-II reaction employs alkynes as substrates. The addition of X radicals to alkynes, followed by 1,5-HAT (hydrogen atom transfer), cyclization, and coupling with Y, affords trifunctionalized products. The Type-III reaction employs alkenes or alkynes as substrates. The addition of X radicals, followed by oxygen trapping of the radical intermediates and enol functionalization with Y, gives the trifunctionalized products. The Type-III reaction could be extended to allenes and enynes. It is noteworthy that three new bonds generated during the trifunctionalization are shown in bold style in the products (Scheme 2). 

**Figure 1:**
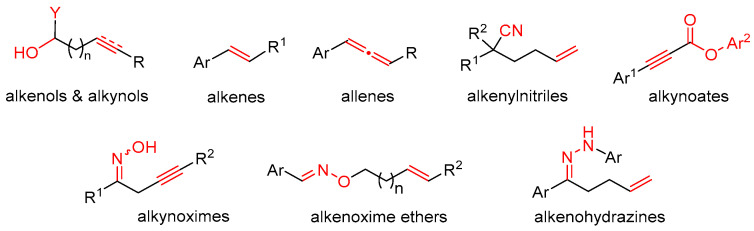
Representative substrates for radical trifunctionalization reactions.

Some popular substrates for trifunctionalization, including alkenols/alkynols, alkenes/allenes, alkenylnitriles, and alkynoates, are shown in Figure 1.

## 2. Reaction of Alkenols and Alkynols

**Scheme 3:**
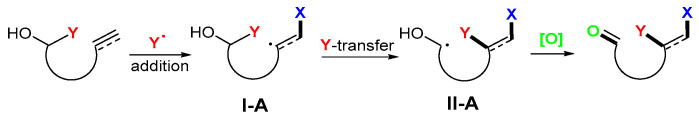
Trifunctionalization of Y-substituted alkenols and alkynols.

Trifunctionalization of alkenols and alkynols are Type-I reactions shown in Scheme 3. They involve the addition of radical Y to form radicals **I-A**, followed by Y group migration to give radicals **II-A**, and the oxidation of OH to the carbonyl group (Scheme 3). In the reaction process, the key step is the Y group transfer. The Y group could be aryl, alkynyl, -CN, -CHO, phosphinoyl (-POR_2_), and sulfonyl (-SO_2_R) groups. It is noteworthy that functional group migration (FGM) could be a rearrangement process involving radical cyclization and cleavage. In the case of aryl migration, it is an *ipso*-cyclization/cleavage reaction process. 

### 2.1. Reaction of Alkenols

**Scheme 4:**
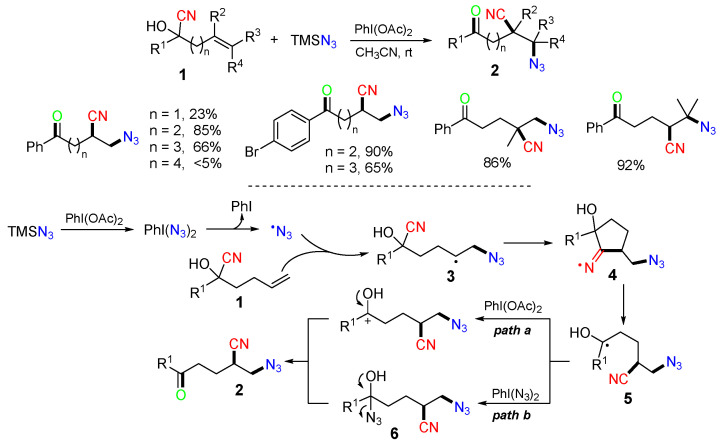
Reaction of alkenyl cyanohydrins involving CN shift.

The Zhu group is one of the pioneers who made contributions to the trifunctionalization of alkenols and alkynols [36,37]. They reported the first example of the trifunctionalization of alkenyl cyanohydrins in 2016 (Scheme 4) [38]. The key step of CN-transfer could be 1,3- to 1,6-shifts, but only 1,4- and 1,5-shifts give meaningful yields. 

The mechanism proposed in Scheme 4 suggested that the azido radical generated from the interaction of trimethylsilyl azide (TMSN_3_) with PhI(OAc)_2_ adds to the C=C bond of alkenyl cyanohydrins **1** to afford alkyl radicals **3**, which then shifts the CN group through cyclic iminyl radicals **4** to form radicals **5**. There could be two ways to convert radicals **5** to products **2:** via oxidation with PhI(OAc)_2_ or through the formation of azidohydrins **6**.

**Scheme 5:**
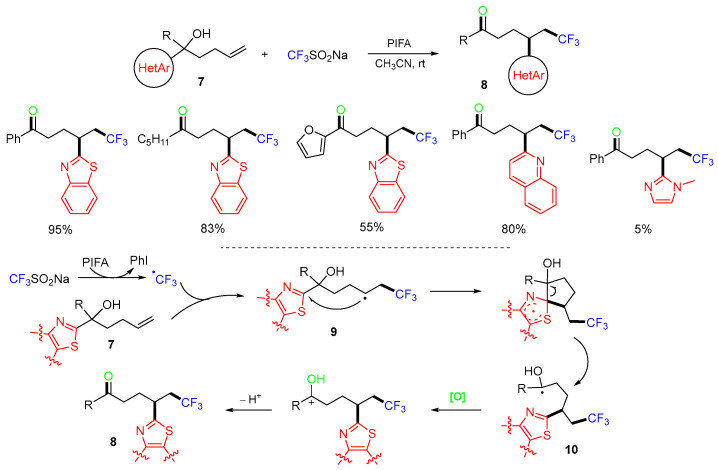
Reaction of heteroaryl alkenols involving heteroaryl shift.

The Zhu group reported a method for distal heteroaryl shift in 2017 (Scheme 5) [39]. This method is good for 1,4-aryl shift since the migration via 5-membered *ipso*-radical intermediates is more favorable than other ring sizes. In the reaction process, a CF_3_ radical generated from the *Langlois’ reagent* (CF_3_SO_2_Na) and phenyliodine bis(trifluoroacetate) (PIFA) add to the C=C bond of hydroxy-substituted alkenes **7** to give alkyl radical intermediates **9**, which then undergo 1,4-shift of the aryl group to form more stable hydroxyalkyl radicals **10**, followed by oxidization to give products **8**.

**Scheme 6:**
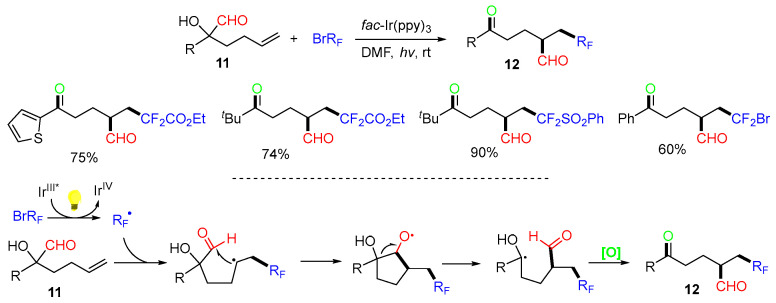
Reaction of *α*-carbonyl tertiary alcohols involving carbonyl shift.

The Zhu group extended the reaction scope for carbonyl migration (Scheme 6) [40]. The photoredox reaction of *α*-formyl-substituted alkenols **11** with fluoroalkyl bromides afforded formyl-migrated trifunctional products **12**. 

**Scheme 7:**
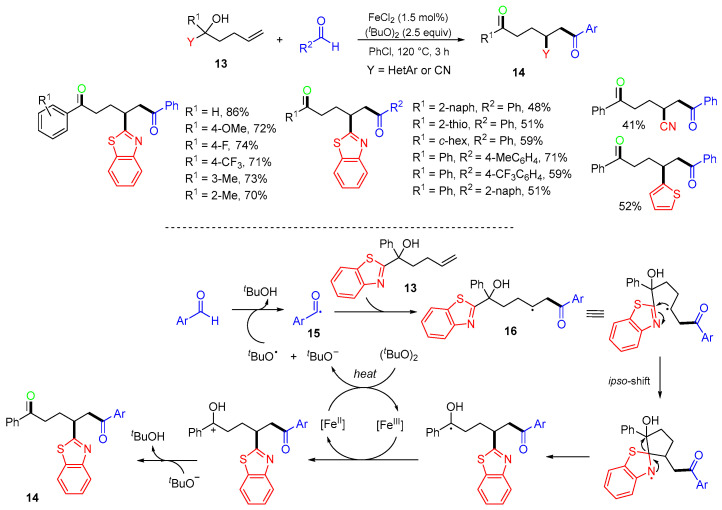
Fe-catalyzed reaction of alkenols involving heteroaryl migration.

A Fe^II^-catalyzed reaction of alkenols **13** involving heteroaryl group shift to give 1,6- and 1,7-diketons **14** was reported by the Li group in 2020 (Scheme 7) [41]. The proposed mechanism suggested that the *^t^*BuO radical generated from di-*tert*-butyl peroxide (DTBP) in the presence of Fe^II^ abstracts hydrogen from aldehydes to afford acyl radicals **15**. The addition of acyl radicals to the alkene moiety of alkenols **13** produces alkyl radicals **16**, followed by intramolecular radical *ipso*-migration and Fe^III^-catalyzed oxidation to give products **14**. 

**Scheme 8:**
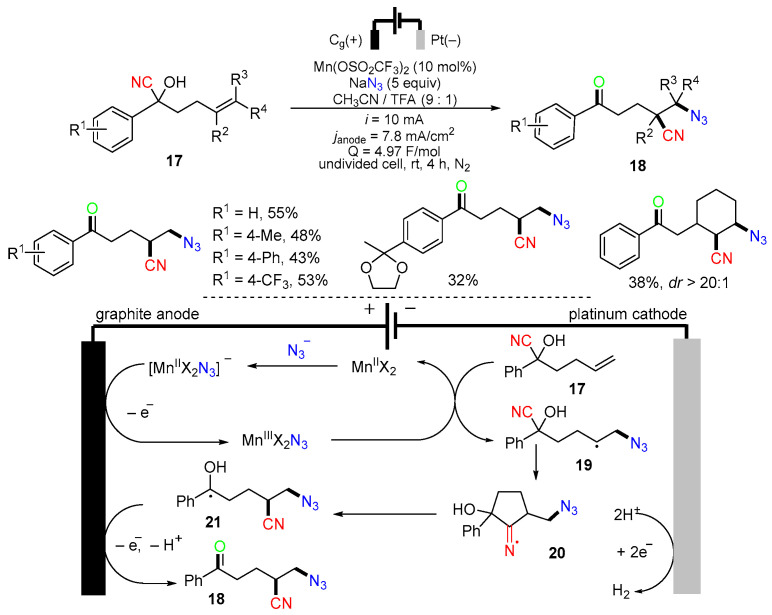
Electrochemical reaction alkenyl cyanohydrins involving 1,4-CN migration.

An electrochemical reaction of alkenyl cyanohydrins **17** for making azidocyanated products **18** was reported by the Morrill group in 2022 (Scheme 8) [42]. The proposed mechanism suggested that the [Mn^II^X_2_N_3_]^−^ generated from the interaction of Mn^II^X_2_ with NaN_3_ is oxidized at the graphite anode to give Mn^III^X_2_N_3,_ which delivers the azido radicals. The addition of azido radicals to alkene **17** to give secondary alkyl radicals **19**, followed by cyclization, affords cyclic iminyl radicals **20**. *β*-Scission of radicals **20** gives *α*-hydroxy alkyl radicals **21**, which are oxidized at the anode to give final products **18**. Hydrogen gas evolves through proton reduction at the cathode.

**Scheme 9:**
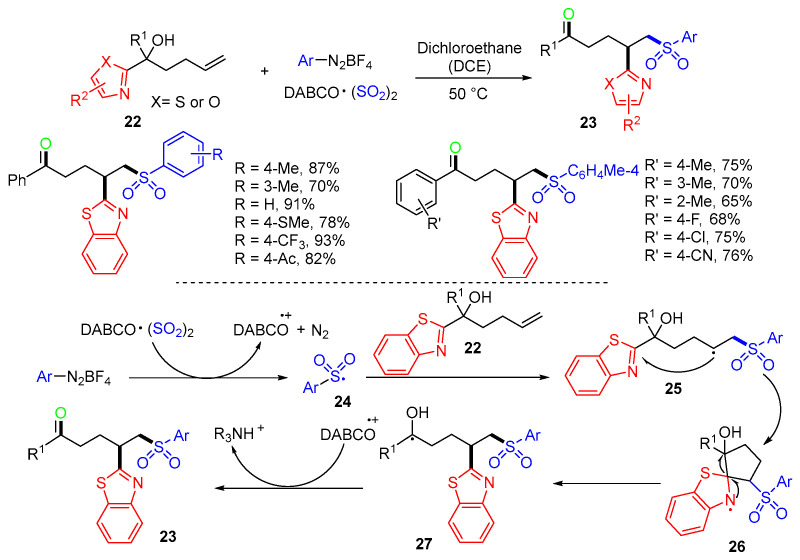
Reaction of alkenols involving heteroaryl shift.

A three-component reaction of heteroaryl-substituted alkenols **22**, aryldiazonium tetrafluoroborates, and 1,4-diazabicyclo [2.2.2]octane (DABCO)·(SO_2_)_2_ was reported by Wu’s group in 2021 [43]. Under catalyst-, base-, and additive-free conditions, the reaction gave products **23** in moderate to good yields (Scheme 9). The proposed mechanism suggested that sulfonyl radicals **24** generated from aryldiazonium tetrafluoroborate and DABCO·(SO_2_)_2_ undergo radical addition to alkenols **22** to afford carbon radicals **25**, followed by cyclization to give the radicals **26**. Ring-opening of radicals **26** forms more stabilized radicals **27**, followed by oxidation to give products **23**.

**Scheme 10:**
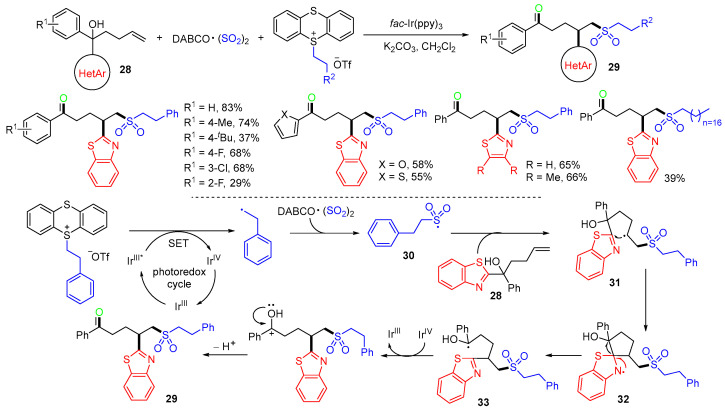
Photoredox reaction of alkenols involving heteroaryl shift.

Very recently, the Guo group reported a photoredox reaction of alkenols **28**, DABCO·(SO_2_)_2,_ and thianthrenium salts for the synthesis of alkylsulfonylated ketones **29** (Scheme 10) [44]. In the reaction process, phenethyl radicals generated from the thianthrenium are captured by SO_2_ from DABCO·(SO_2_)_2_ to generate phenethylsulfonyl radicals **30**. The addition of radicals **30** to the terminal C=C bond carbon of tertiary alcohol **28** affords intermediates **31**, which go through a five-membered cyclic transition state to give spiro *N*-radical **32**. The ring opening of unstable spiro structures **32** gives more stable hydroxyalkyl radicals **33**, which are oxidized to give final products **29** after deprotonation.

**Scheme 11:**
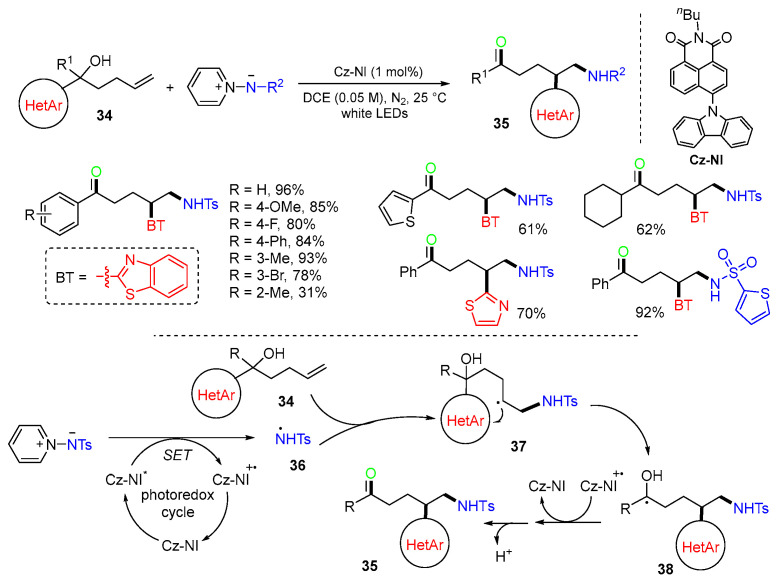
Photoredox reaction of alkenols involving heteroaryl shift.

A photoredox reaction for the synthesis of amino ketones was reported by the Xu group in 2022 [45]. The Cz-NI-catalyzed reaction of alkenols **34** with *N*-protected 1-aminopyridinium under LED irradiation afforded distal amino ketones **35** in moderate to excellent yields (Scheme 11). In the reaction process, the *N*-centered radical **36** generated from the *N*-protected 1-aminopyridinium adds to the C=C bond of hydroxyl-substituted alkenes **34** to give alkyl radical intermediates **37**, which then undergo 1,4-shift of the heretoaryl group to give more stable hydroxyalkyl radicals **38**, followed by oxidization and deprotonation to give the final products **35**.

**Scheme 12:**
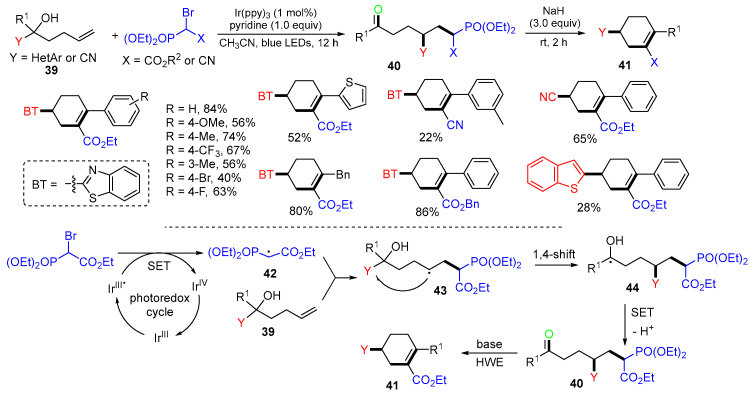
Synthesis of intramolecular HWE reaction.

The Feng group, in 2021, reported a straightforward photoredox reaction of alkenols **39** for the preparation of phosphonylated ketones **40**, which undergo spontaneous Horner−Wadsworth−Emmons (HWE) olefination under a basic condition to give compounds **41** (Scheme 12) [46]. In the reaction process, single-electron transfer (SET) oxidation of bromophosphonates by photoexcited *fac*-Ir(ppy)_3_ generates radical **42**, followed by addition to the terminal alkene of alkenols **39** to provide alkyl radical intermediates **43**. The Y group 1,4-shift of radical **43** gives more stable hydroxyalkyl radicals **44**, which are oxidized to give final products **40** after deprotonation.

**Scheme 13:**
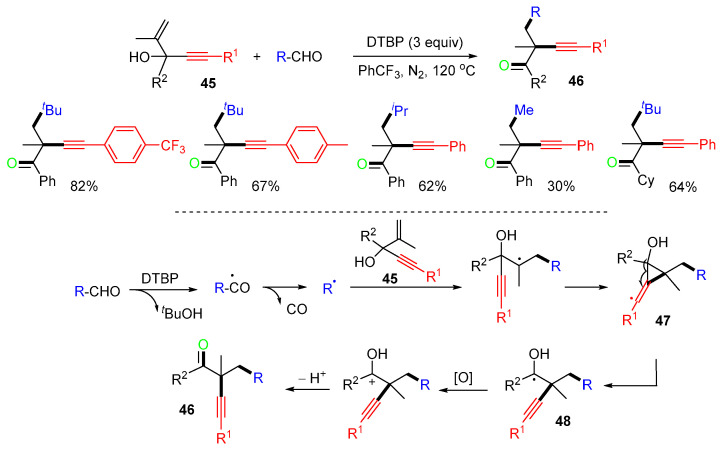
Reaction of 1,4-enyn-3-ols with alkylaldehydes.

The reaction of 1,4-enyn-3-ols **45** with alkylaldehydes for the synthesis of *α*,*β*-functionalized ketones **46** was reported by the Xiang group in 2021 (Scheme 13) [47]. Alkyl radicals, generated from the decarbonylation of alkylaldehydes using DTBP as an oxidant, add to the C=C bond of 1,4-enyn-3-ols **45**, followed by an alkynyl shift through the three-membered ring intermediates **47** to give radicals **48**. Oxidation of **48** with DTBP and deprotonation give products **46**. A similar reaction for the synthesis of *α*-alkynyl ketones was reported by the Tu group [48].

**Scheme 14:**
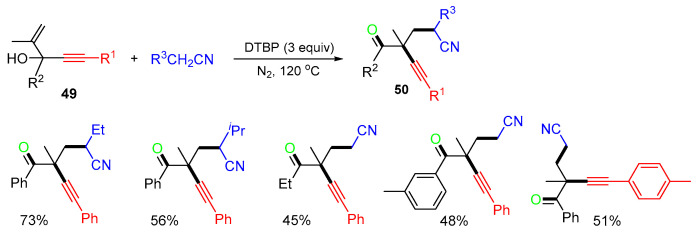
Reaction of 1,4-enyn-3-ols with alkyl nitriles.

The reaction of 1,4-enyn-3-ols **49** with nitriles for the synthesis of cyanolated ketones **50** was reported by Cheng and coworkers in 2021 (Scheme 14) [49]. The alkyl radicals generated from the reaction of nitriles using DTBP as an oxidant afforded products **50** in moderate yields.

**Scheme 15:**
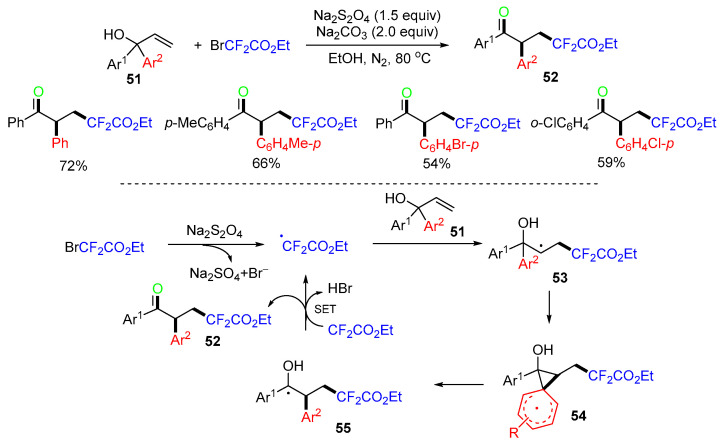
Reaction of allylic alcohols with ethyl bromodifluoroacetate.

The Chu group, in 2021, reported a reaction of diaryl allylic alcohols involving 1,2-aryl migration [50]. The reaction of 1,1-diarylprop-2-en-1-ols **51** and BrCF_2_CO_2_Et in the presence of sodium hydrosulfite as a radical initiator afforded products **52** in moderate to good yields (Scheme 15). In the reaction process, the CF_2_CO_2_Et radical generated from BrCF_2_CO_2_Et adds to the C=C bond of diaryl allylic alcohols **51** to form the alkyl radicals **53**. Then, 1,2-aryl migration of **53** via the spiro[2,5]octadienyl radical intermediates **54** leads to the *α*-hydroxy carbon-centered radicals **55**, which undergo a SET process to produce the final products **52** along with the regeneration of the CF_2_CO_2_Et radical. Some related methods for the radical trifunctionalization of diaryl allylic alcohols via 1,2-aryl migration by metal catalysis could be found in recent reviews [51,52].

**Scheme 16:**
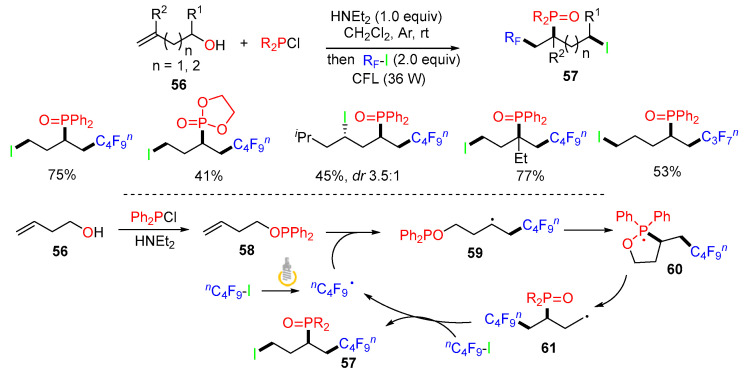
Reaction of alkenols involving phosphinoyl group shift.

The Han group introduced a trifunctionalization reaction involving a phosphinoyl group shift in 2022 [53]. The reaction of homoallylic alcohols **56**, R_2_PCl, and fluoroalkyl iodides (R_F_-I) under the irradiation of visible light afforded products **57** in good to excellent yields (Scheme 16). The proposed mechanism suggested that the *^n^*C_4_F_9_ radical generated from *^n^*C_4_F_9_-I adds to the C=C bond of **58** to afford alkyl radicals **59**. Radical cyclization onto the phosphine affords cyclic phosphine radicals **60**, followed by *β*-fragmentation of the C–O bond to form *C*-radicals **61**, which are trapped by *^n^*C_4_F_9_-I through an iodine-atom transfer process to give products **57**.

**Scheme 17:**
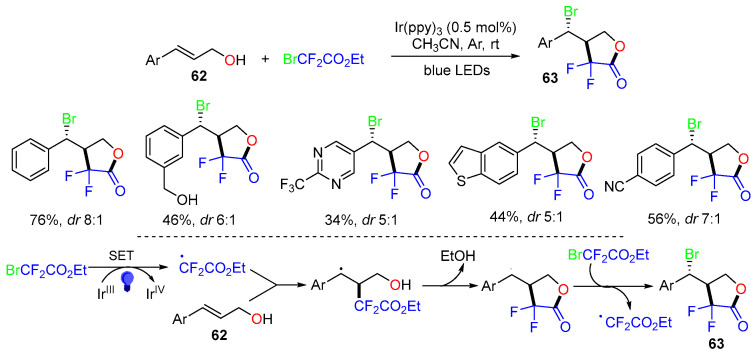
Trifunctionalization of cinnamyl alcohols with ethyl bromodifluoroacetates.

In 2022, Molander and coworkers reported a photoredox reaction of cinnamyl alcohols **62** with ethyl bromodifluoroacetate under blue LED irradiation to give brominated *α,α*-difluoro-*γ*-lactones **63** in moderate to good yields in a diastereoselective manner (Scheme 17) [54]. In the reaction process, the CF_2_CO_2_Et radical generated from BrCF_2_CO_2_Et adds to the C=C bond of cinnamyl alcohols **62**, followed by lactonization and the Br-atom transfer process to give products **63**.

**Scheme 18:**
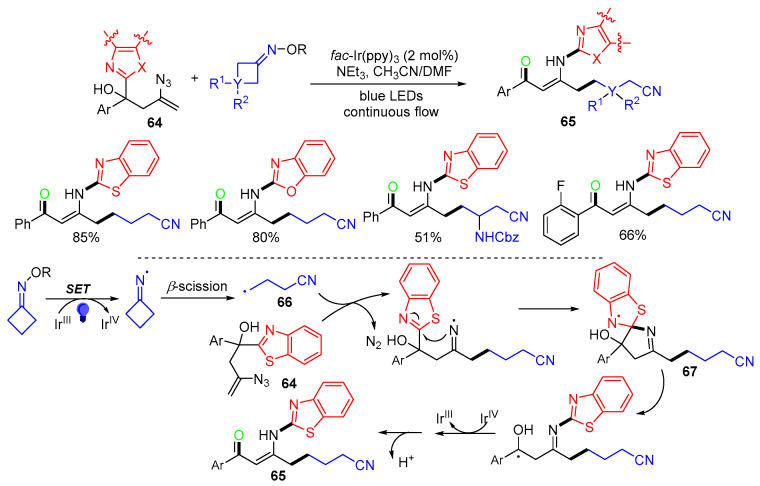
Reaction of azidyl homoallylic alcohols with cycloketone oxime esters.

A photoredox reaction of heterocyclic-substituted azidyl homoallylic alcohols **64** with cycloketone oxime esters was reported by the Guo group in 2021 [55]. The reaction via 1,4-heteroaryl migration from the *C*-center to the *N*-center affords a variety of cyanoalkylacylated *β*-enamino ketone **65** in moderate-to-good yields under continuous-flow conditions (Scheme 18). The reaction process involves the addition of cyanoalkyl radical **66** to the C=C bond of homoallylic alcohols **64** with the loss of N_2_, followed by the *C*-center to the *N*-center heteroaryl migration through the key spiro radicals **67**, single electron oxidation, and further deprotonation to give products **65**.

**Scheme 19:**
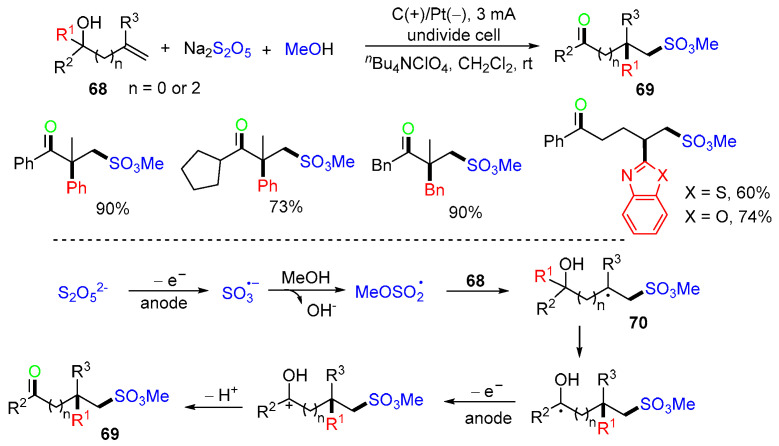
Electrochemical reaction of alkenols with Na_2_S_2_O_5_ and MeOH.

In 2022, the Ye group introduced an electrochemical reaction of alkenols **68** with Na_2_S_2_O_5_ and MeOH for the synthesis of ketosulfonate esters **69** (Scheme 19) [56]. The sulfite radical anion, generated from the anode oxidation of Na_2_S_2_O_5,_ is trapped by MeOH to afford the methoxysulfonyl radical, which then adds to the C=C bond of alkenols **68** to yield radicals **70**. Functional group migration (FGM), anodic SET oxidation, and deprotonation afford products **69**.

### 2.2. Reaction of Alkynols

Alkynol-based radical reactions are presented in this section. The addition of a radical to the double bond of alkenols generates alkyl radical intermediates, while the addition of a radical to the triple bond of alkynols generates more reactive vinyl radical intermediates, which could be more favorable for FGM as well as HAT in the trifunctionalization reactions.

**Scheme 20:**
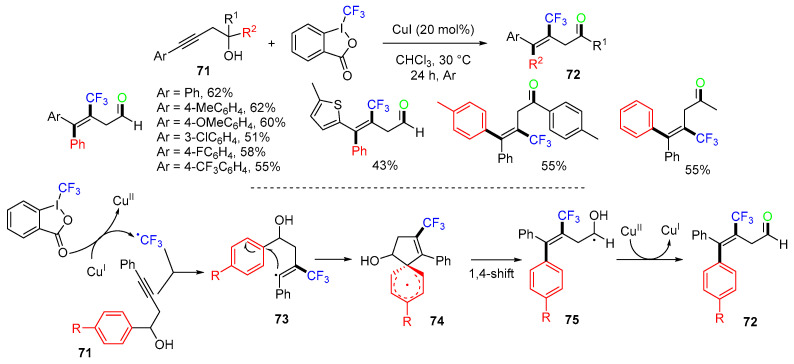
Cu-catalyzed reaction of alkynols involving 1,4-aryl migration.

In 2014, Liang’s group reported a Cu-catalyzed reaction of homopropargylic alcohols **71** with *Togni’s II reagent* for the synthesis of CF_3_-containing 3-buten-1-one derivatives **72** (Scheme 20) [57]. The proposed mechanism suggested that the CF_3_ radical, generated from the reaction of *Togni’s II reagent* and Cu^I^ species, adds to the alkyne of homopropargylic alcohols **71** to give radicals **73**, followed by 5-*ipso* cyclization to form radicals **74**, and then radicals **75** for 1,4-aryl migration. Finally, single-electron oxidation of radical **75** with Cu^II^ species furnishes product **72** and a regenerated Cu^I^ catalyst.

**Scheme 21:**
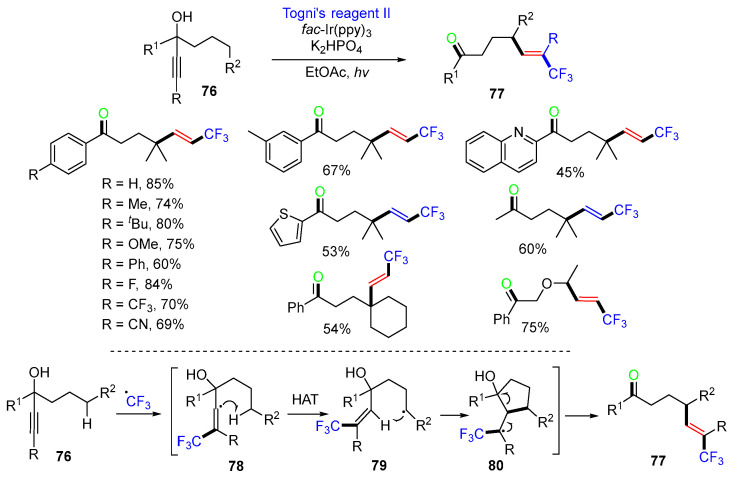
Regioselective reaction of alkynols involving 1,5-HAT.

In 2019, the Zhu group reported a CF_3_ radical addition-initiated reaction of alkynols **76** for the synthesis of CF_3_-containing 5-hexen-1-ones **77** under mild photoredox reaction conditions (Scheme 21) [58]. The proposed mechanism suggested that the addition of CF_3_ radicals to alkynols **76** generates alkenyl radicals **78**, which undergo 1,5-HAT to produce alkyl radicals **79**. Cyclization of alkyl radicals **79** affords cyclopentanol intermediates **80**, which undergo C–C bond cleavage to give stereoselective *E*-olefins **77**.

**Scheme 22:**
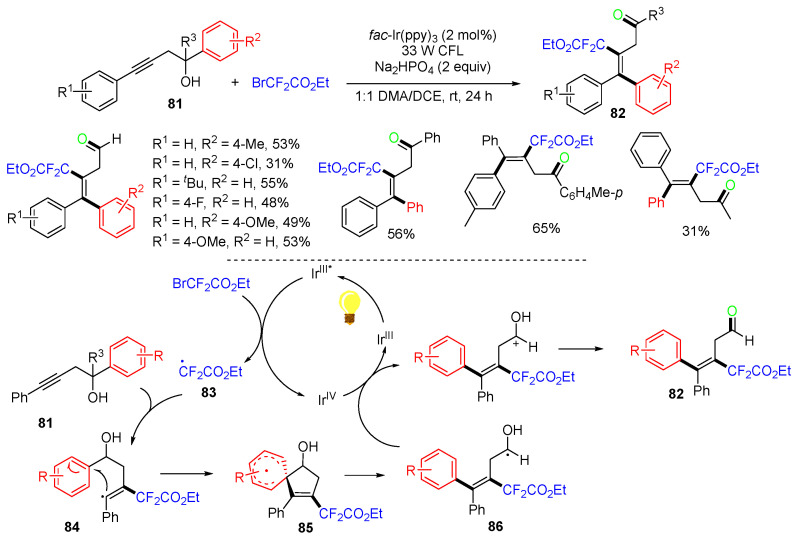
Reaction of homopropargylic alkenols involving 1,4-aryl migration.

The Zhu group, in 2017, reported a carbodifluoroalkylation reaction of homopropargylic alcohols **81** under photoredox conditions (Scheme 22) [59]. In this reaction, BrCF_2_CO_2_Et is used as a source for difluorination. The proposed mechanism suggested that the active state Ir^III*^ generated from photocatalyst *fac*-Ir(ppy)_3_ reacts with BrCF_2_CO_2_Et through a SET to give radical **83**, which adds to the alkyne moiety of homopropargylic alcohols **81** to give radicals **84**, followed by 5-*ipso* cyclization and cleavage of radicals **85** to give hydroxyalkyl radicals **86**. SET oxidation and then deprotonation of radicals **86** give products **82**.

**Scheme 23:**
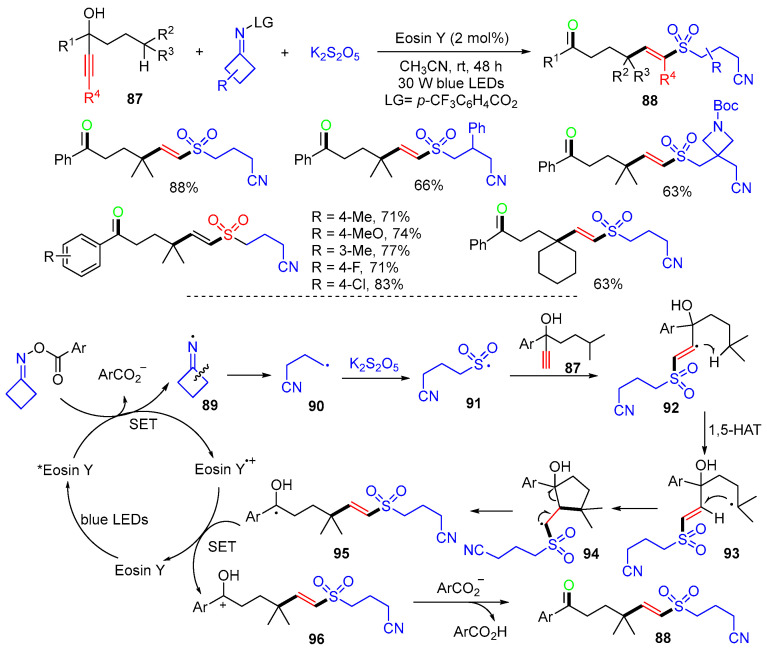
Reaction of alkenols involving 1,4-vinyl migration.

A photoredox reaction of propargyl alcohols **87**, K_2_S_2_O_5_, and cycloketone oxime esters for the synthesis of cyanoalkylated vinyl sulfone **88** was reported by the Wu group in 2021 (Scheme 23) [60]. The initial step of the synthesis is the irradiation of Eosin Y under blue light to form excited Eosin Y*, which undergoes SET with cycloketone oxime ester to form iminyl radical **89** and then cyanoalkyl radical **90** after the C–C bond cleavage. The reaction of radical **90** with K_2_S_2_O_5_ leads to the formation of cyanoalkylsulfonyl radical **91**, which adds to propargyl alcohols **87** to form radicals **92**. 1,5-HAT of vinyl radicals **92** results in alkyl radicals **93**, which undergo cyclization to give radicals **94**. The C–C bond cleavage of **94** gives vinyl-migrated radicals **95**, which undergo SET with Eosin Y•^+^ to give carbocations **96** and then products **88** after deprotonation.

### 2.3. Reaction of Alkenol Derivatives

Other than the reactions of alkenols and alkynols presented above, alkenol derivatives could be used for the trifunctionalization reactions.

**Scheme 24:**
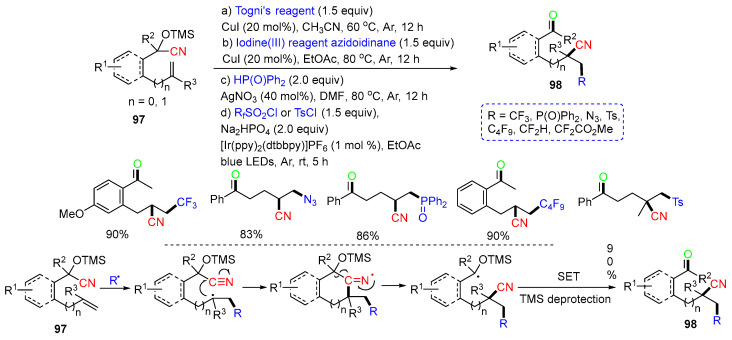
Reaction of TMS-protected alkenyl cyanohydrins involving CN migration.

In 2016, the Liu group reported the CN migration reaction of TMS-protected alkenyl cyanohydrins **97**. The reaction process involves the addition of R radicals (CF_3_, N_3_, P(O)Ph_2_, C_4_F_9_, and Ts radicals), remote CN group migration, and converting of OTMS to carbonyl group to afford cyano ketones **98** (Scheme 24) [61].

**Scheme 25:**
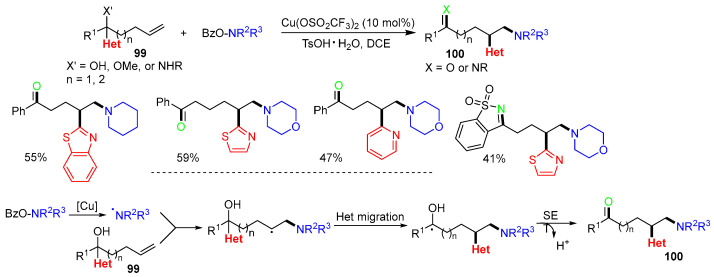
Reaction of functionalized alkenes involving heterocyclic group migration.

A Cu-catalyzed aminoheteroarylation of alcohol-, amino-, and ether-containing alkenes **99** was reported by the Liu group in 2021 [62]. The second functional group, X, was derived from X’ (OH, OMe, or NHR). The reaction process involves the addition of amino radicals generated from the BzONR^2^R^3^ to the C=C bond of alkene **99**, followed by heteroaryl migration, SET, and deprotonation to afford the final product **100** (Scheme 25).

**Scheme 26:**
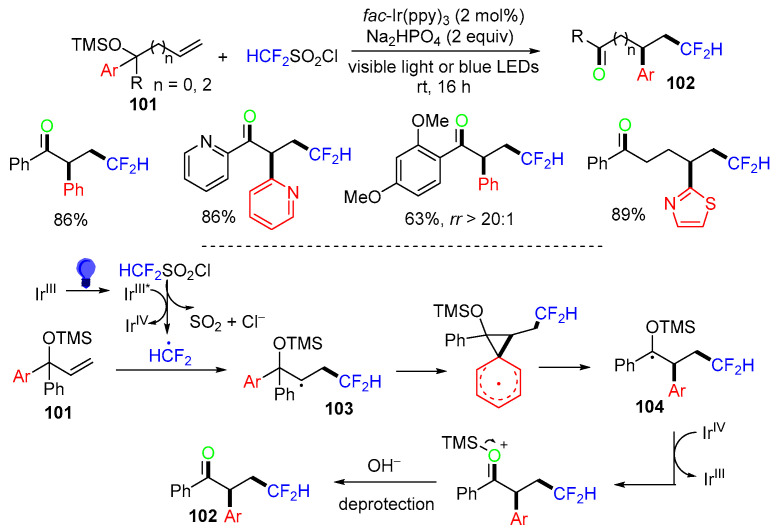
Reaction of TMS-protected alkenols involving Ar migration.

A photoredox-catalyzed reaction of *α*-aryl allyl alcohol TMS ethers **101** for the preparation of difluoromethyl-containing ketones **102** was reported by the Dolbier group in 2022 (Scheme 26) [63]. The proposed mechanism suggested that the CF_2_H radical derived from HCF_2_SO_2_Cl adds to the terminal carbon of alkenes **101** to form radical **103**, followed by aryl *ipso*-migration to give radicals **104** and Ir-catalyzed oxidative SET to afford products **102** after deprotection. It is noteworthy that if there are two aryl groups that could undergo migration, the *rr* is used to indicate the selectivity of two aryl groups.

**Scheme 27:**
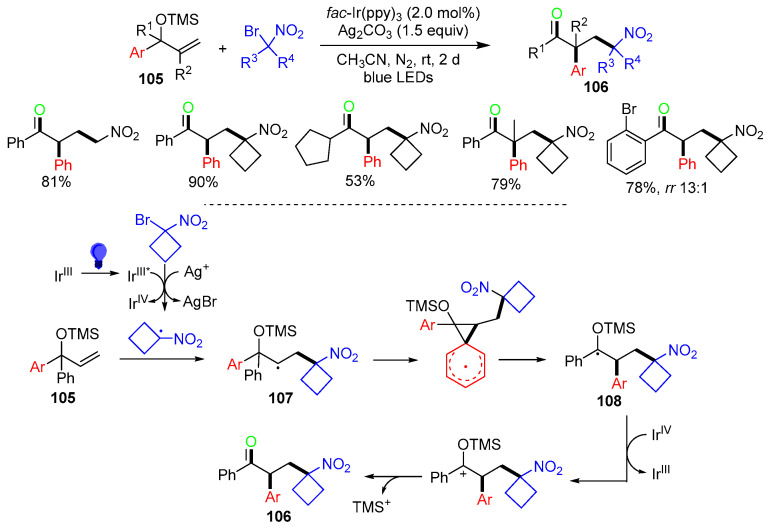
Reaction of *α*-aryl allyl alcohol TMS ethers.

A photoredox reaction of *α*-aryl allyl alcohol TMS ethers **105** with *gem*-bromonitroalkanes for the synthesis of *α*-aryl-*γ*-nitro ketones **106** was reported by the Jiao group in 2023 (Scheme 27) [64]. The reaction process involves the addition of *α*-nitrocyclobutyl radicals, generated from the reaction of *gem*-bromonitrocylcobutane with photoexcited *fac*-^*^Ir(ppy)_3_ and Ag^+^ to the C=C bond of **105**, to generate radicals **107**, which undergo aryl *ipso*-migration to give α-trimethylsilyloxy radical **108**, followed by SET oxidation to give products **106** after deprotection of TMS.

**Scheme 28:**
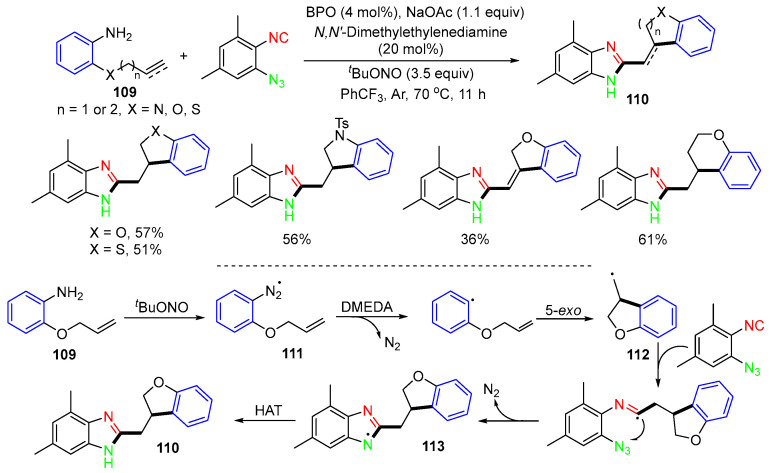
Reaction of 2-(allyloxy)-aniline for the synthesis of 2-substituted benzoimidazoles.

A radical reaction of alkenyl anilines **109** and 1-azido-2-isocyanoarenes involving denitrogenative radical cyclization for making 2-substituted benzoimidazoles **110** was reported by the group in 2017 (Scheme 28) [65]. The reaction process involves the generation of azidal radicals **111**, releasing nitrogen gas, followed by radical cyclization to form radicals **112**. Radicals **112** add to 1-azido-2-isocyano-3,5-dimethylbenzene, followed by denitrogenative cyclization to form benzoimidazole radicals **113**, and then products **110** after HAT.

## 3. Reaction of Alkenes and Allenes

**Scheme 29:**
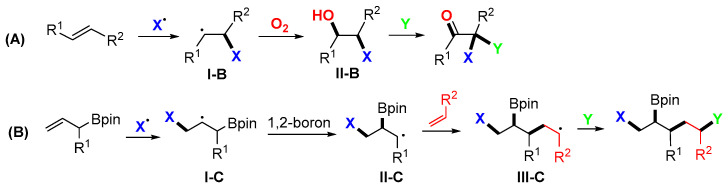
Representative pathways for trifunctionalization of alkenes.

The trifunctionalization reaction of non-activated alkenes generally undergoes the Type-III pathway shown in Scheme 2. The initial radical addition gives the intermediate radicals **I-B**, which are oxidized to carbon cations and then react with O_2_, H_2_O, RCN, or other species to introduce the second and third functional groups (Scheme 29A). The reaction of functionalized alkenes could be designed for 1,2-group migration (1,2-GM), such as that shown in Scheme 29B. In the racial addition of allenes, a radical added to the terminal carbon of allenes generates vinyl radicals (like the reaction of alkynes), while a radical added to the middle carbon of allenes generates alkyl radicals (similar to the reaction of alkenes).

### 3.1. Reaction of Alkenes

**Scheme 30:**
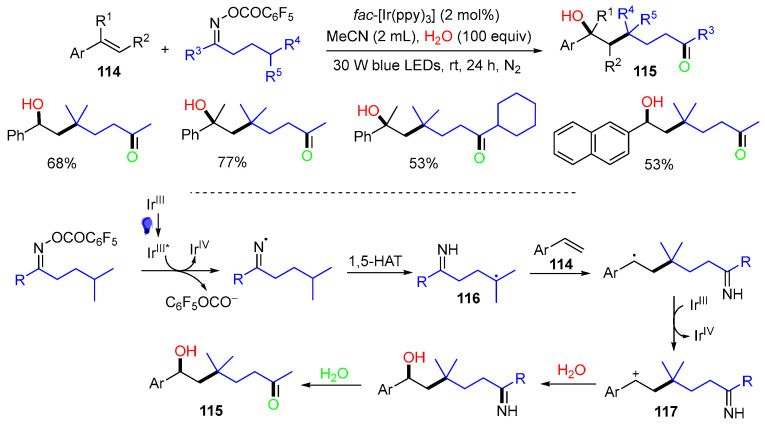
Reaction of aryl alkenes with oxime esters.

A visible-light-induced reaction of alkenes **114** with oxime esters for making functionalized ketones **115** was reported by Ma and coworkers in 2018 (Scheme 30) [66]. The reaction involves the generation of iminyl radicals, which are rearranged to alkyl radicals **116** via 1,5-HAT. The first functionalization of alkene **114** is the addition of alkyl radicals **116**, followed by SET oxidation to cations **117**, which react with H_2_O, followed by the hydrolysis of the emine group to afford products **115**.

**Scheme 31:**
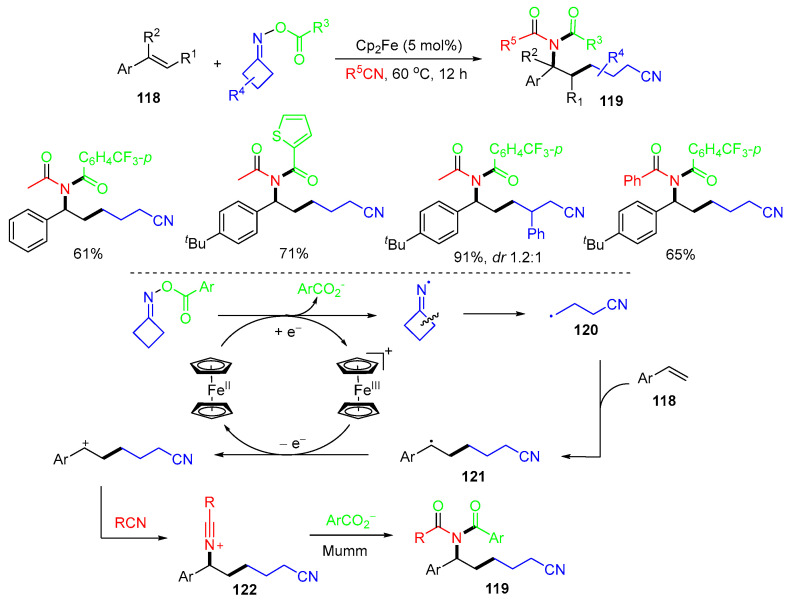
Reaction of alkenes with cycloketone oxime esters.

Another iminyl radical-initiated reaction of alkenes **118** was reported by the Golagani group in 2023 (Scheme 31) [67]. Under the catalysis of ferrocene, homolytic N–O bond cleavage of cycloketone oxime esters gives cyclic iminyl radicals, which convert to the CN-alkyl radical **120** after ring opening. The addition of the radical **120** to the terminal position of the aryl alkene **118** gives radicals **121**, followed by SET oxidization and then nucleophilic attack by nitriles to give **122**, which undergo Mumm rearrangement to give products **119**.

**Scheme 32:**
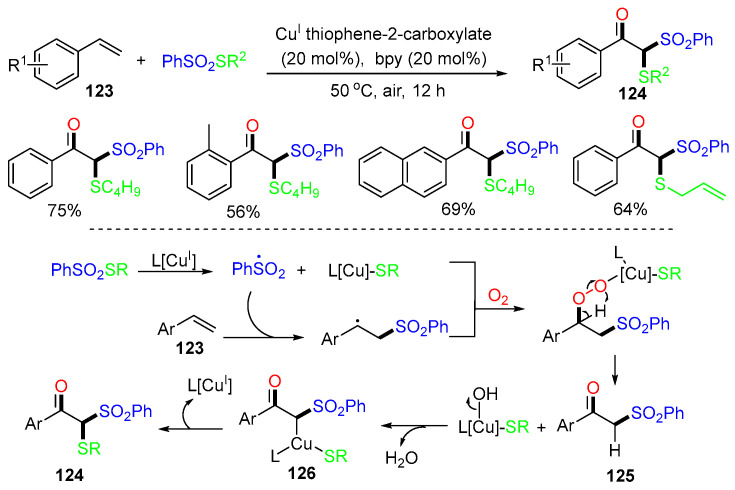
Reaction of styrenes for making *S*-alkyl benzenesulfonothioates.

In 2018, the Xu group introduced a Cu-catalyzed reaction of alkenes **123** for the synthesis of functionalized *β*-keto thiosulfones **124**. The arylsulfonyl radical derived from PhSO_2_SR adds to styrenes **123**, followed by Cu-assisted oxidation with O_2_ gives **125**. Formation of Cu-complex **126** followed by reductive elimination of L[Cu^I^] affords products **124** (Scheme 32) [68].

**Scheme 33:**
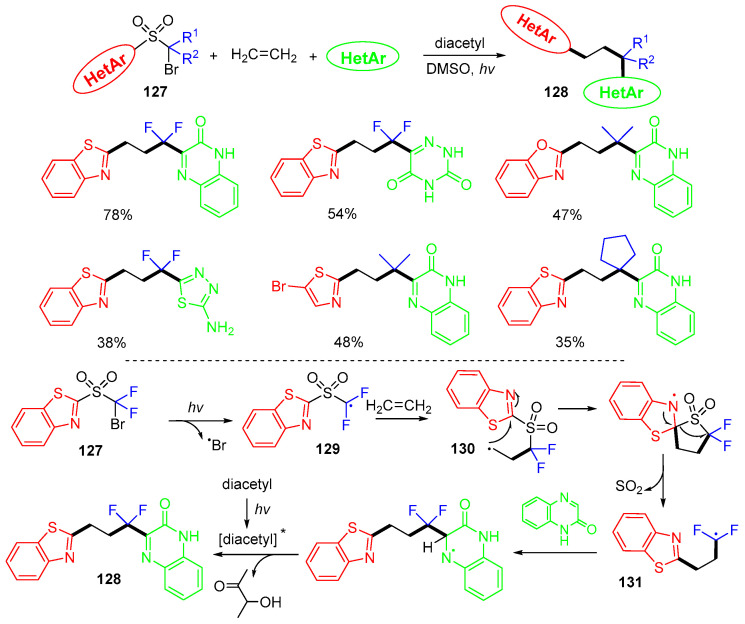
Reaction of ethylene involving desulfonylative 1,2-heterocyclic group migration.

A method for the reaction of ethylene **127** with bifunctional sulfonyl reagents and electron-deficient heterocyclic compounds for the synthesis of diheteroarylated products **128** was reported by the Zhu group in 2023 (Scheme 33) [69]. In the reaction process, difluoromethyl radicals **129** derived from a sulfonyl reagent are added to ethylene **127** to afford alkyl radical **130**, which undergoes desulfonylative 1,2-heterocyclic group migration via *ipso*-rearrangement to give **131**. The Minisci-type addition of **131** to quinoxalinone affords product **128** after rearomatization.

### 3.2. Reaction of Functionalized Alkenes

**Scheme 34:**
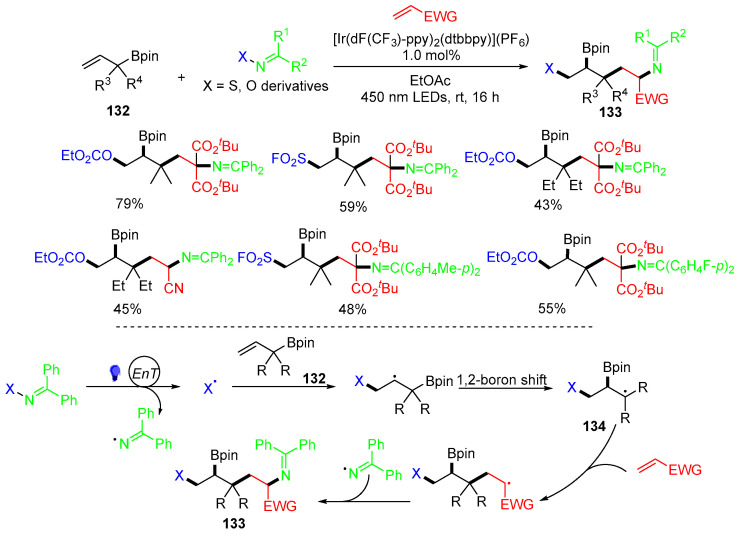
Reaction of alkenes involving 1,2-boron shift.

Other than the Type-III radical trifunctionalization reaction of alkenes shown above, there are a couple of special cases involving the 1,2-group migration. In 2023, the Glorius group reported a reaction involving 1,2-boron shift of alkenes **132** and imine-based bifunctional reagents for making 1,2,5-trifunctionalized products **133** (Scheme 34) [70]. Under photoredox conditions, mine-based reagents release X and iminyl radicals. The X radicals add to allylboronic esters **132**, followed by a 1,2-boron shift to form radicals **134**. Then, the addition of radicals **134** to the second alkenes, followed by coupling with an iminyl radical, gives products **133**.

**Scheme 35:**
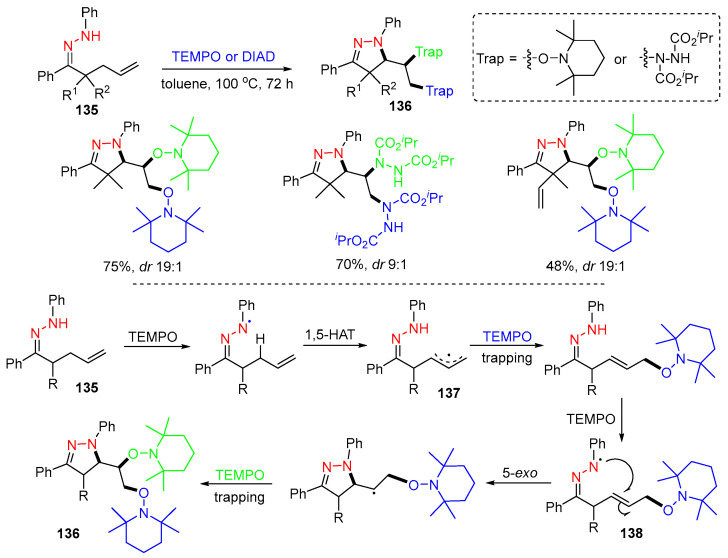
Reaction of alkenyl hydrazones with TEMPO or DIAD.

Duan and coworkers, in 2013, reported a reaction of hydrazones **135** with radical trapping agents TEMPO (2,2,6,6-tetramethyl-1-piperidinyloxy) or DIAD (diisopropyl azodicarboxylate) to afford functionalized pyrazolines **136** with good stereoselectivity (Scheme 35) [71]. In the reaction involving TEMPO, initial hydrazone radicals are generated by the reaction of hydrazone **135** with TEMPO. The radicals undergo 1,5-HAT to form allyl radicals **137**, which are trapped by TEMPO, followed by hydrogen atom abstraction with TEMPO to generate second hydrazone radicals **138**. The 5-*exo-trig* cyclization of **138** generates pyrazoline-containing radicals, which are trapped by TEMPO to give products **136**. It is noteworthy that TEMPO is used as a hydrazone radical initiator as well as a carbon radical scavenger.

**Scheme 36:**
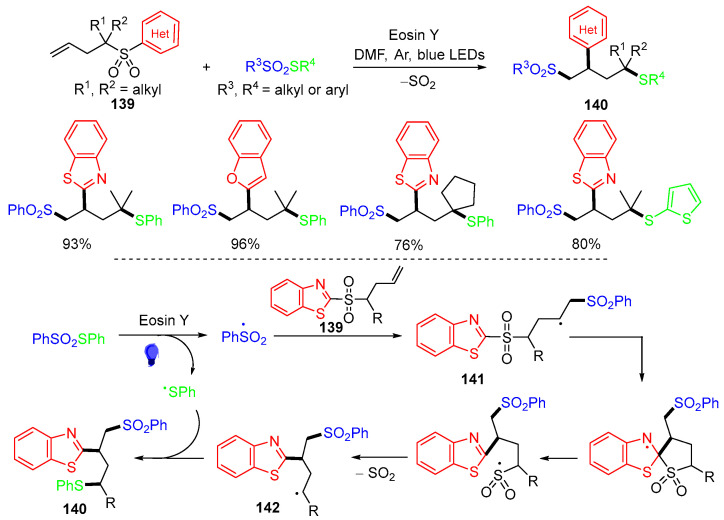
Reaction of butenyl benzothiazole sulfones and analogs.

The Wang group, in 2021, reported a visible-light-promoted radical sulfonyl–Smiles rearrangement reaction of butenyl benzothiazole sulfone **139** with thiosulfonates or selenosulfonates for making 1,2,4-trifunctionalized butane products **140** (Scheme 36) [72]. The proposed mechanism suggested that the sulfonyl radical derived from thiosulfonates adds to the terminal double bond of butenyl benzothiazole sulfones **139** to form intermediate radicals **141**. *Ipso*-radical cyclization of **141** followed by ring-opening and desulfonylation afford heteroaryl group migrated radicals **142**. Trapping **142** by the phenylthio radical affords products **140**. Other heterocyclic groups containing butenyl sulfones could be used for this reaction [73].

**Scheme 37:**
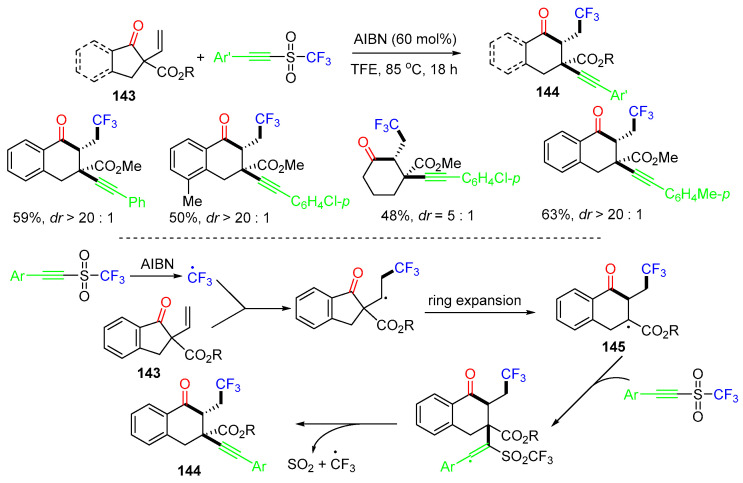
Reaction of *α*-vinyl-*β*-ketoesters.

A radical 1,2,3-tricarbofunctionalization of *α*-vinyl-*β*-ketoesters was reported by the Bao group in 2022. The reaction of *α*-vinyl-*β*-ketoesters **143** and alkynyl triflones in the presence of azodiisobutyronitrile (AIBN) as the radical initiator afforded trifunctional products **144** with excellent diastereoselectivity (Scheme 37) [74]. The reaction process involves the addition of a CF_3_ radical to the C=C bond of α-vinyl-β-ketoesters **143**, followed by ring expansion to form radicals **145**. The addition of **145** to alkynyl triflones, followed by the elimination of SO_2_ and CF_3_ radicals, gives products **144**.

**Scheme 38:**
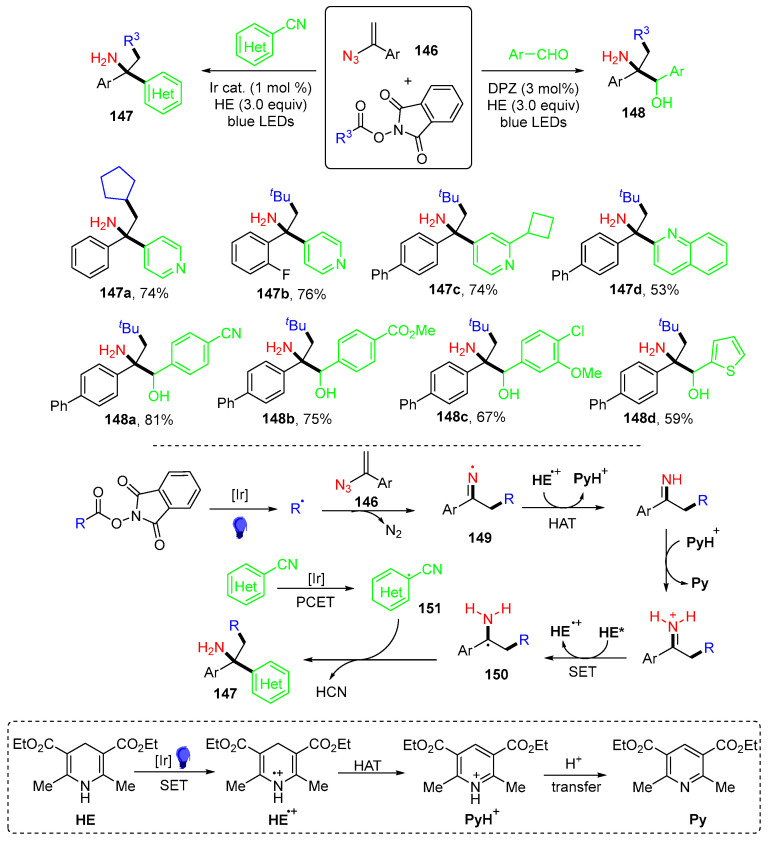
Reaction of vinyl azides and NHPI with cyanoarenes or aryl aldehydes.

The Shu group, in 2024, introduced a set of reactions of vinyl azides **146** and *N*-hydroxyphthal-imide (NHPI) with cyanoarenes esters for the synthesis of *α*-tertiary primary amines **147** or with aryl aldehydes for *α,α’,α’*-trisubstituted primary amines **148** (Scheme 38) [75]. In the reaction involving cyanoarenes, alkyl radicals generated from NHPI under photoredox catalysis are added to vinyl azides to afford the iminyl radicals **149** after denitrogenation. HAT of **149**, followed by proton transfer from PyH^+^ and SET reduction afford radicals **150**. Meanwhile, the reduction of cyanoarenes occurs through a proton-coupled electron transfer (PCET) process to give persistent radicals **151**. The coupling of **150** with **151** and the elimination of HCN result in products **147**.

### 3.3. Reaction of Allenes

The addition of a radical to allenes could happen at the terminal carbon of allenes to generate vinyl radicals (similar to the reaction of alkynes) or at the middle carbon of allenes to generate alkyl (allylic) radicals (similar to the reaction of alkenes). Two examples in this section involve the inter- and intramolecular radical addition of allenes.

**Scheme 39:**
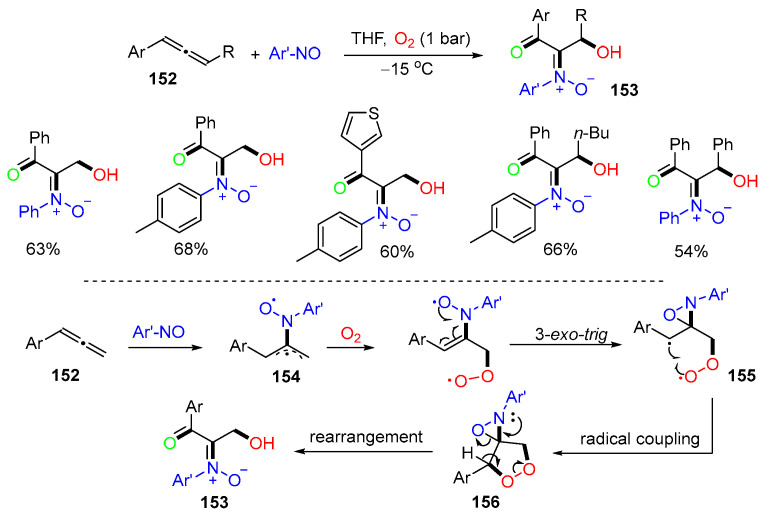
Reaction of allenes for *O,N,O*-trifunctionalization.

A reaction for *O,N,O*-trifunctionalization of allenes **152** with nitrosoarenes and oxygen was reported by the Liu group in 2017. The reaction was carried out at −15 °C to afford 3-hydroxy-1-ketonyl-2-imine oxides **153** in good yields (Scheme 39) [76]. The proposed mechanism suggested that nitrosobenzene adds to the arylallene **152** to form 1,4-diradicals **154**, followed by the capture of oxygen and 3-*exo-trig* cyclization to form diradicals **155**. Intramolecular radical coupling of **155** affords dioxygen-containing oxacycle **156** and then product **153** after ring-opening rearrangement.

**Scheme 40:**
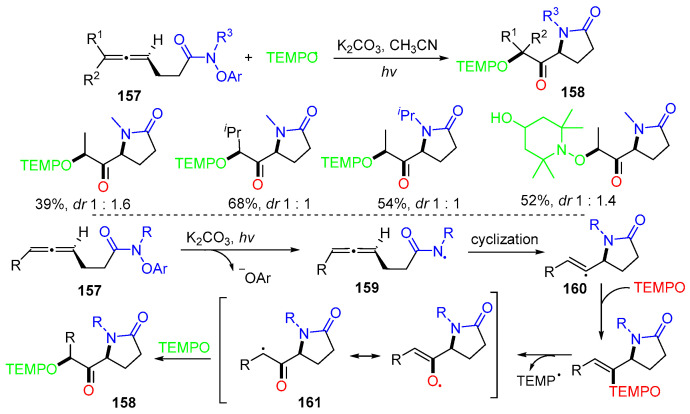
Reaction of aryloxyamino-containing allenes with TEMPO derivatives.

A photo-induced radical reaction of aryloxyamino-containing allenes **157** with TEMPO derivatives (R_2_N-O^●^) for the synthesis of TEMPO-substituted lactams **158** was reported by the Schomaker group in 2021 [77]. The reaction was carried out under 440 nm light irradiation in the presence of K_2_CO_3_ (Scheme 40). In the reaction process, the *N*-centered radicals **159** derived from aryloxyamino-containing allenes **157** add to the proximal C=C bond of allene **157** to generate vinyl radicals **160**, which are trapped by TEMPO, followed by the elimination of TEMP to afford radicals **161**, which are coupled with TEMPO again to form products **158**.

## 4. Reaction of Alkynes and Enynes

**Scheme 41:**
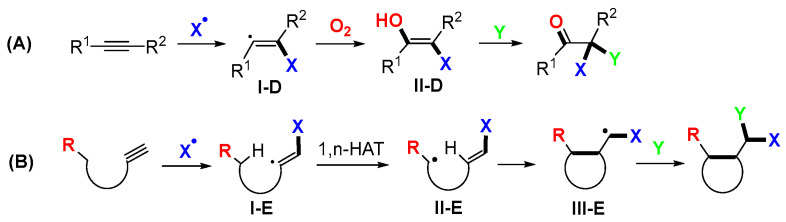
Two kinds of trifunctionalization reactions of alkynes.

Depending on the substrate structures and reaction conditions, alkynes could undergo trifunctionalization reactions without group migration of intermediate radicals (Scheme 41A) or involving 1,5-HAT, as shown in Scheme 41B.

### 4.1. Reaction of Alkynes

**Scheme 42:**
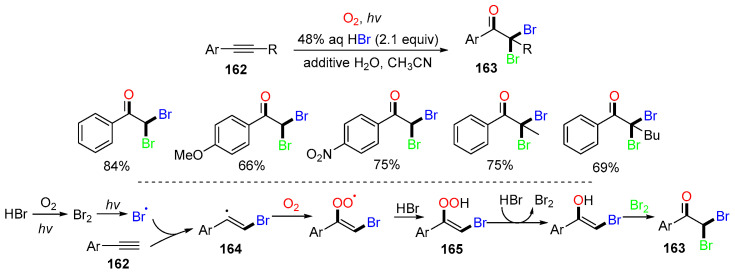
Reaction of aryl alkynes with HBr and oxygen.

A photo-oxidative trifunctionalization of aryl alkynes was reported by the Itoh group in 2010 [78]. The reaction of aromatic alkynes **162** and HBr (aq.) in the presence of molecular oxygen afforded *α,α*-dibromoacetophenones **163** in good yields (Scheme 42). The proposed mechanism suggested that the Br radical, formed under visible light irradiation of HBr in the presence of O_2_, adds to phenylacetylenes **162** to generate vinyl radicals **164**, which are oxidized by oxygen, followed by the H atom abstraction from HBr and reduction with HBr to provide bromoenols **165**. Trapping of Br_2_ by **165** affords dibromoacetophenone **163**.

**Scheme 43:**
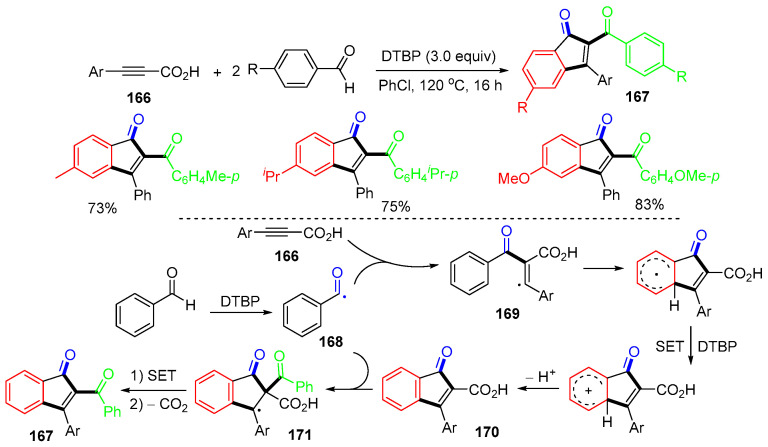
Reaction of phenylpropiolic acids with benzaldehydes.

A reaction of phenylpropiolic acids **166** with benzaldehydes using DTBP as an oxidant for the synthesis of 2-aroyl-3-phenylindenones **167** was reported by the Jafarpour group in 2020 (Scheme 43) [79]. The *α*-carbonyl radicals **168**, generated from the reaction of aldehydes with DTBP, add to phenylpropiolic acids **166** to afford vinyl radicals **169**, which undergo radical aromatic substitution, SET oxidation, and deprotonation to afford indenone-2-carboxylic acids **170**. The addition of radicals **168** to **170** affords radicals **171**, which undergo SET oxidation and decarboxylation to give products **167**.

**Scheme 44:**
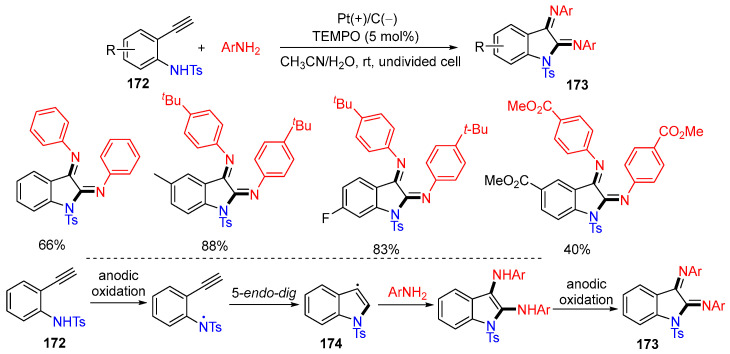
Electrochemical triamination of alkynes with anilines.

In 2022, the Huang group reported an electrochemical reaction using TEMPO as the redox catalyst for the triamination of aryl alkynes **172** with anilines for the synthesis of functionalized indolines **173** (Scheme 44) [80]. The *N*-centered radicals generated from aryl alkynes **172** undergo 5-*endo-dig* cyclization to give radicals **174**, followed by couplings with anilines and the oxidation on the anode to give products **173**.

**Scheme 45:**
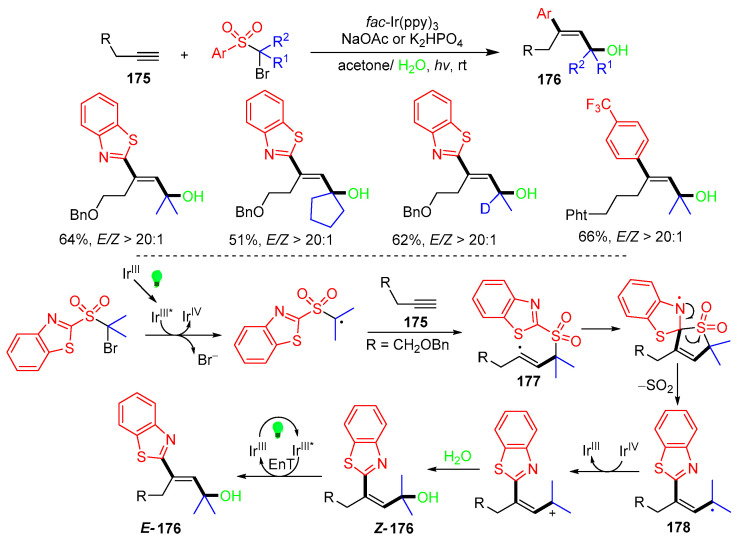
Reaction of aliphatic alkynes with sulfones.

In 2024, the Zhu group reported a photoredox reaction of aliphatic alkynes **175** with sulfones for the synthesis of functionalized *E*-allyl alcohol **176** (Scheme 45) [81]. The reaction processes involve the formation of the initial alkyl radical for addition to alkynes **175** to form vinyl radicals **177**, followed by the *ipso*-cyclization of **177** and desulfonylative ring-opening to give 1,2-heterocyclic group-migrated radicals **178**. SET oxidation of **178** is followed by trapping with H_2_O to afford *Z*-allyl alcohols **176**, which are isomerized to *E*-allyl alcohols **176**.

**Scheme 46:**
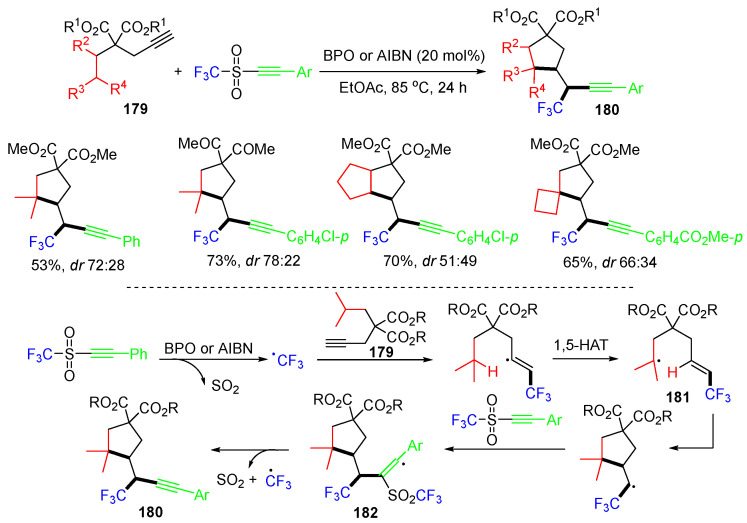
Reaction of alkynes with alkynyl triflones involving 1,5-HAT.

The Studer group, in 2020, introduced a 1,1,2-trifunctionalization of terminal alkynes involving 1,5-HAT of intermediate vinyl radicals. The reaction of alkynyl triflones with alkynes **179** in the presence of dibenzoyl peroxide (BPO) or AIBN as the radical initiator furnished the substituted cyclopentanes **180** in moderate to good yields (Scheme 46) [82]. The reaction process involves the addition of the CF_3_ radical to alkynes **179**, followed by 1,5-HAT to form radicals **181**. Cyclization of **181** and trapping with alkynyl triflones afford radicals **182**. Products **180** are produced after the fragmentation of the CF_3_SO_2_ group from **182**.

**Scheme 47:**
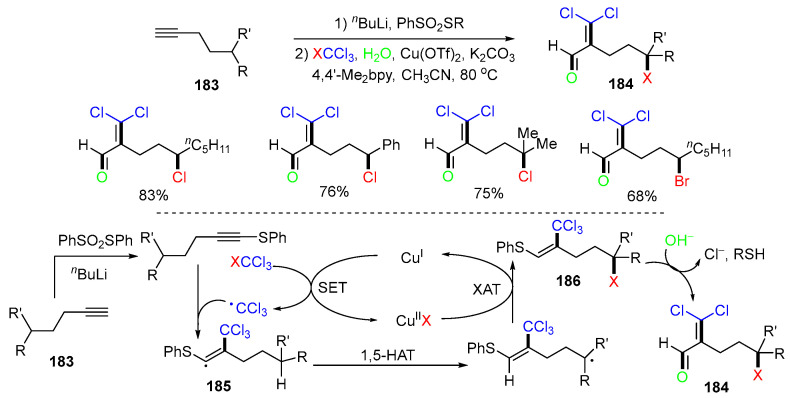
Reaction of alkynes using SPh as the transient directing group.

Another Cu-catalyzed 1,2,5-trifunctionalization reaction of alkynes **183** involving 1,5-HAT and using the SPh group as the transient directing group was reported by the Zhu group in 2022 to afford functionalized aldehydes **184** in good to excellent yields (Scheme 47) [83]. The proposed mechanism suggested that the electrophilic CCl_3_ radical, generated from SET oxidation of XCCl_3_ (X = Cl or Br), attacks at the C2 position of RS-attached alkynes that were generated from **183** to give RS-attached vinyl radicals **185**, which undergo 1,5-HAT and Cu-assisted halogen atom transfer to give **186**, followed by S_N_2′-type substitution and elimination of the PhS group and Cl anion to form products **184**.

### 4.2. Reaction of Enynes

**Scheme 48:**
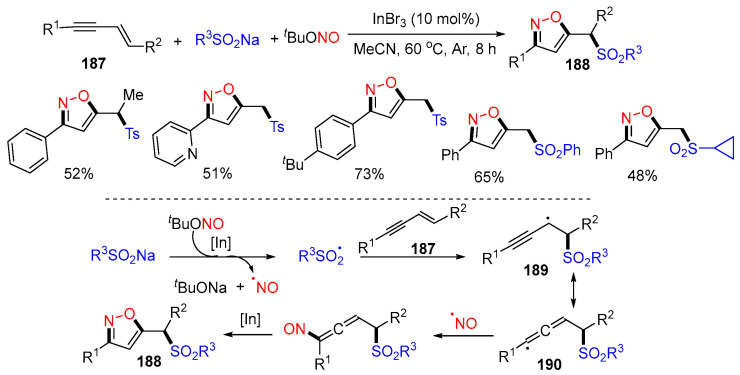
Trifunctionalization of 1,3-enynes with sodium sulfinates and TBN.

In 2020, the Li group reported an In-catalyzed [3+2] annulation of 1,3-enynes **187** to form functionalized isoxazoles **188** (Scheme 48) [84]. In the reaction process, the sulfonyl radicals derived from sodium sulfinates add to the C=C bond of enynes **187** to give sulfonyl-containing alkyl radicals **189**, which have resonance structures of **190**. Radical cross-coupling of **190** with NO radical, followed by isomerization and intramolecular nucleophilic annulation, affords products **188**.

## 5. Reaction of Alkenylnitriles

**Scheme 49:**
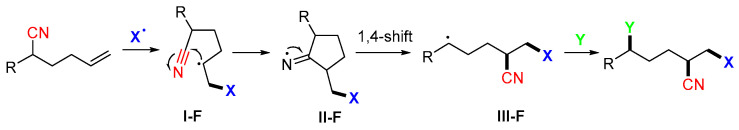
General pathway for the reaction of alkenylnitriles.

Alkenes bearing a CN group at *γ*-position are good substrates for trifunctionalizations since the CN group could be relocated via 1,4-migration after the radical addition (Scheme 49). The CN group migration involves the formation of radicals **I-F**, the cleavage of cyclic iminyl radicals **II-F**, and the 1,4-shift to radicals **III-F**.

**Scheme 50:**
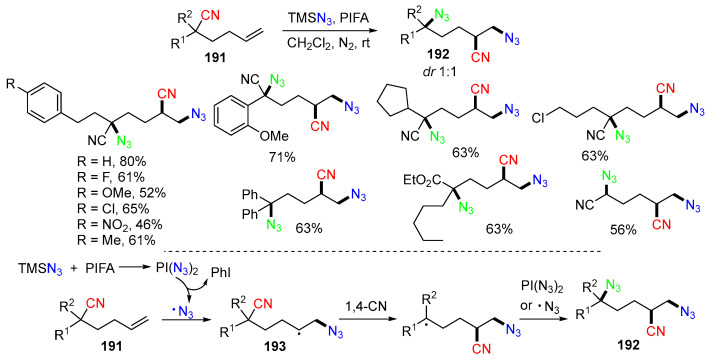
Reaction of hexenenitriles involving 1,4-CN migration.

A trifunctionalization reaction of hexenenitriles **191** with TMSN_3_ was reported by the Zhu group in 2022 (Scheme 50) [85]. In this synthesis, the N_3_ radical generated from the reaction of TMSN_3_ and PIFA adds to hexenenitriles **191** to form alkyl radicals **193**, which undergo 1,4-CN group migration followed by trapping the second N_3_ group to give products **192**. The evaluation of the chain length for the CN group migration indicated that the 1,5-CN migration also readily proceeded to give the desired product. Meanwhile, the 1,3-CN migration only gave a trace amount of the products.

**Scheme 51:**
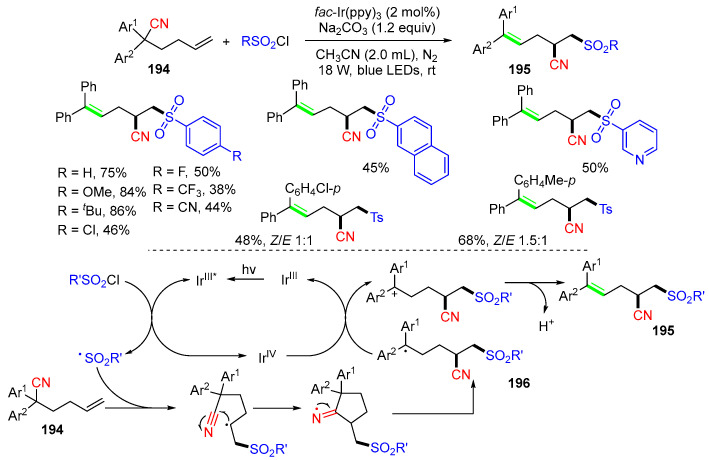
Reaction of hexenenitriles with aryl sulfonyl chlorides involving 1,4-CN migration.

The Zhu group, in 2023, reported another hexenenitrile-based reaction for the synthesis of trisubstituted alkenes (Scheme 51) [86]. The reaction of hexenenitriles **194** with aryl sulfonyl chlorides under blue LED irradiation and in the presence of *fac*-Ir(ppy)_3_ at room temperature gave products **195** in moderate to good yields. The proposed mechanism suggested that the excited Ir^III*^ species interacts with sulfonyl chlorides to form sulfonyl radicals, which add to hexenenitriles **194**, followed by 1,4-CN migration to give stabilized benzyl radicals **196**. SET oxidation of radicals **196** with Ir^IV^, followed by base-promoted deprotonation, gives products **195**.

**Scheme 52:**
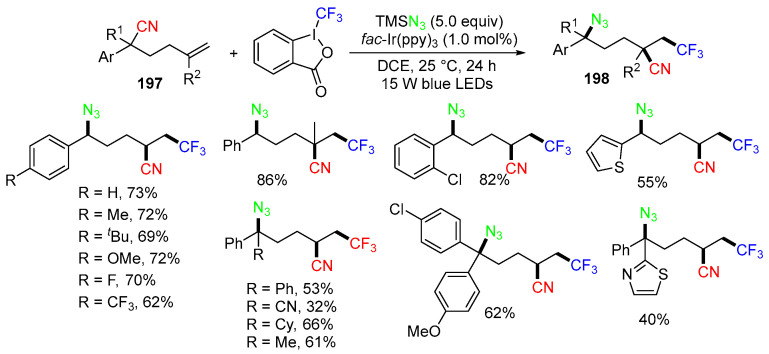
Reaction of hexenenitriles with Tongi’s reagent II involving 1,4-CN migration.

In 2022, Chen and Zhu’s groups reported a photoreaction system of hexenenitriles **197** using *Tongi’s reagent II* as a radical source for making 1,2,5 trifuctionalized alkanes **198** (Scheme 52) [87]. Other than the formation of CF_3_ radicals, the reaction mechanism is similar to that shown in Scheme 51.

**Scheme 53:**
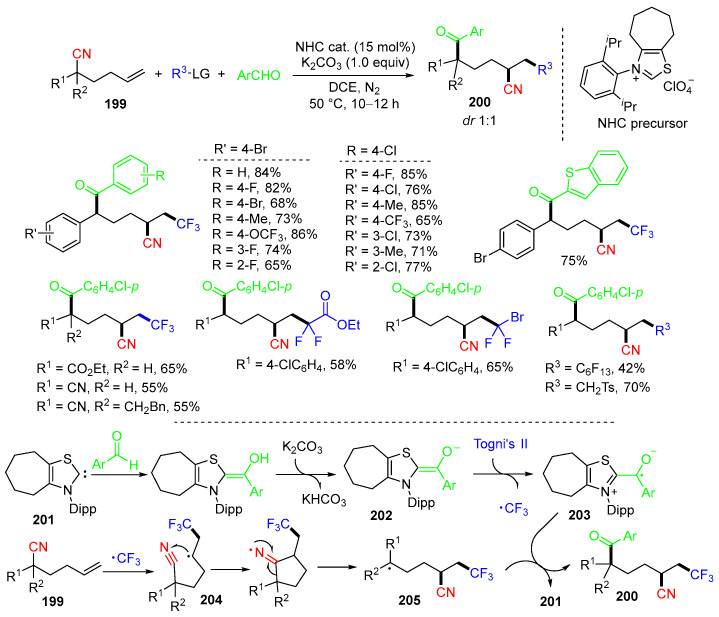
NHC-catalyzed reaction of hexenenitriles.

Very recently, Du and coworkers developed an *N*-heterocyclic carbene organocatalytic reaction of hexenenitriles **199** with *Tongi’s reagent II* and aldehydes for making functionalized ketones **200** (Scheme 53) [88]. The proposed mechanism suggested that the reaction of aldehydes and NHC **201** gives Breslow intermediates **202**, which interact with *Togni’s reagent II* to afford NHC-bound ketyl radicals **203** and CF_3_ radicals. The addition of the CF_3_ radical to the C=C bond of hexenenitriles **199** gives alkyl radicals **204**, followed by 1,4-CN migration to give radical **205**, which then couples with the radicals **203** to afford products **200** with the loss of NHC **201** for the next catalytic cycle. The density functional theory (DFT) calculation results showed that the reaction pathway involving 1,4-CN migration is energetically more favorable compared to the pathway without CN migration.

## 6. Reaction of Alkynoates

**Scheme 54:**
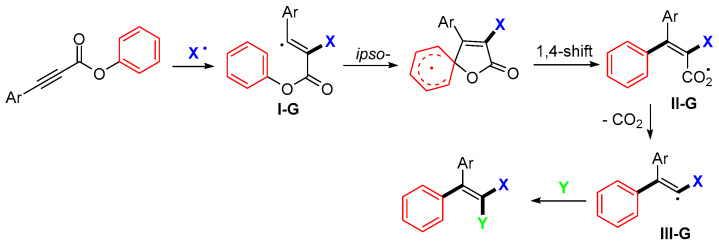
General pathway for the reaction of aryl alkynoates.

Aryl alkynoates bearing two aryl groups are good substrates for trifunctionalization reactions. The red-colored aryl group could be relocated via the processes of *ipso*-cyclization and cleavage (Scheme 54). The Ar group on the terminal carbon of alkyne directs the addition of an X radical to the carbon to form aryl-stabilized vinyl radicals **I-G**, followed by *ipso*-cyclization and cleavage to form radicals **II-G**. The decarboxylated radicals **III-G** are then functionalized by a Y group.

**Scheme 55:**
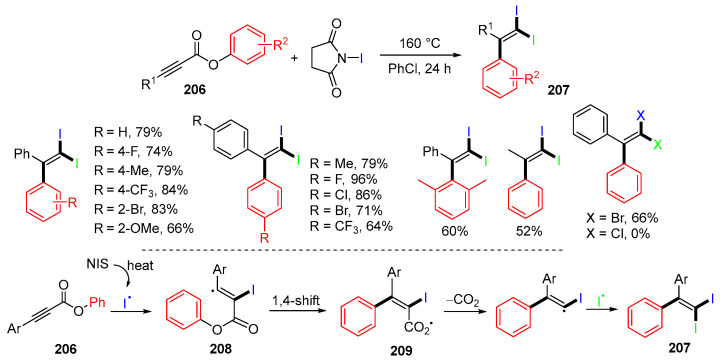
Reaction of alkynoates with NIS involving 1,4-aryl migration.

The Han group, in 2016, reported a reaction of alkynoates **206** for cascade iodination-triggered iodination, aryl migration, decarboxylation, and the second iodination for making functionalized alkenes **207** (Scheme 55) [89]. The proposed mechanism indicated that iodine radicals generated from *N*-iodosuccinimide (NIS) add to the triple bond of alkynoates **206** to afford vinyl radicals **208**, which undergo 1,4-aryl migration to give carboxyl radicals **209**. Decarboxylation of **209** followed by the second iodination gives 1,1-diiodoalkene products **207**. It was found that *N*-bromosuccinimide (NBS), but not *N*-chlorosuccinimide (NCS), could be used to replace NIS for the reaction.

**Scheme 56:**
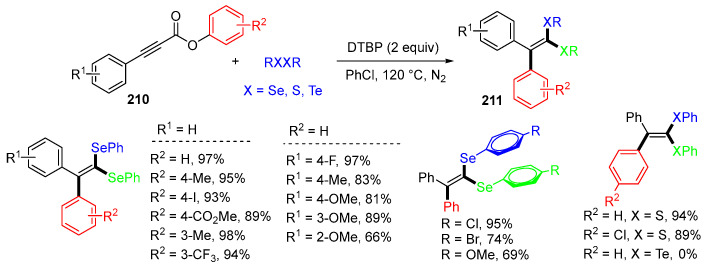
Reaction of alkynoates with dichalcogenides involving 1,4-aryl migration.

The Baidya group, in 2019, modified the reaction conditions and used diphenyl diselenides and other reactants to react with aryl alkynoates **210** to afford tetrasubstituted *gem*-diselenoalkene **211** in good to excellent yields (Scheme 56) [90]. The reaction process is similar to that shown in Scheme 55, which involves the addition of an aryl selenium radical followed by 1,4-aryl migration, decarboxylation, and coupling with a second aryl selenium radical to give products **211**.

**Scheme 57:**
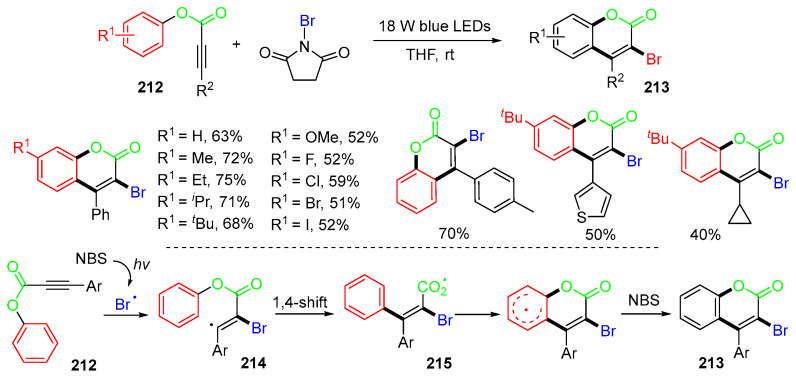
Photo reaction of alkynoates with NBS involving 1,4-aryl migration.

A visible-light-mediated reaction of alkynoates **212** with NBS for the synthesis of 3-bromocoumarins **213** was reported by the She group in 2017 (Scheme 57) [91]. The proposed mechanism suggested that the addition of Br radicals to alkynoates **212** affords vinyl radicals **214**, which undergo 1,4-aryl migration to give carboxyl radicals **215**. Instead of decarboxylation, as shown in Scheme 54, radicals **215** undergo cyclization, followed by aromatization, to afford products **213**. It is noteworthy that under the photoreaction conditions, no decarboxylation of radical **215** occurred, which is different from that shown in Scheme 55.

**Scheme 58:**
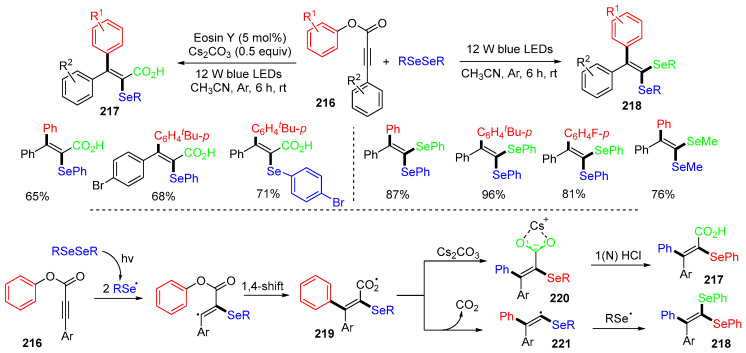
Reaction of aryl alkynoates with diphenyldiselenides.

The Srimani group, in 2022, introduced two photoredox reaction conditions for the trifunctionalization of alkynoates **216** with diphenyl- and dialkyldiselenides (Scheme 58) [92]. The two reaction processes share the same intermediates **219**, which are generated by the addition of selenium radicals to alkynoates followed by 1,4-aryl migration. For the reaction to make products **217**, the Eosin Y photocatalyst transfers an electron to carboxyl radicals **219** to form carboxylate anions **220**, which leads to the formation of products after protonation with HCl. For the reaction to make 1,1-diselenide alkenes **218**, carboxyl radicals **219** undergo decarboxylation to form vinyl radical **221**, followed by coupling with selenium radicals to give the products.

## 7. Reaction of Oximes, Oxime Ethers, and Oxime Esters

**Scheme 59:**
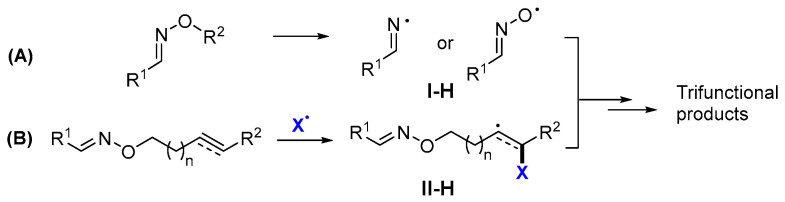
Two pathways for the radical reaction of oximes and derivatives.

Oximes and derivatives have unique =N–O–R functional groups, which readily undergo homolytic cleavage of N–O or O–R bonds to form iminyl or iminoxyl radicals. The iminyl or iminoxyl radicals **I-H** could serve as the initial radicals in the trifunctionalization reactions (Scheme 59A). For oximes tethered with unsaturated carbon bonds, the reaction could be initiated by the addition of radical X to the carbon bond to form radicals **II-H** and then undergo cyclization or group transfer reactions involving the oxime group (Scheme 59B).

**Scheme 60:**
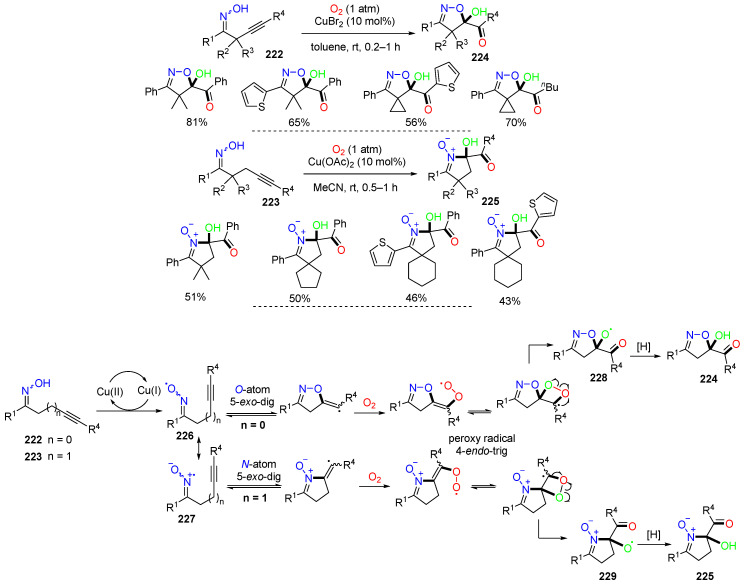
Reaction of *γ,δ*-alkynyl ketoximes.

Han and coworkers, in 2017, reported a Cu-catalyzed dioxygen activation reaction of alkynyl ketoximes **222** and **223** in the synthesis of isoxazoline/cyclic nitrone-featured *α*-ketols **224** and **225** (Scheme 60) [93]. The mechanism investigation revealed that the initial iminoxyl radicals **226** and the resonance structures **227** can undergo dichotomous *O*-/*N*-atom *5*-*exo*-*dig* cyclization to the alkyne group, followed by oxygen activation, peroxy radical *4*-*endo*-*trig* cyclization, and O–O bond cleavage to give oxy radicals **228** or **229**, which leads to the formation of products **224** or **225** after hydrogen abstraction.

**Scheme 61:**
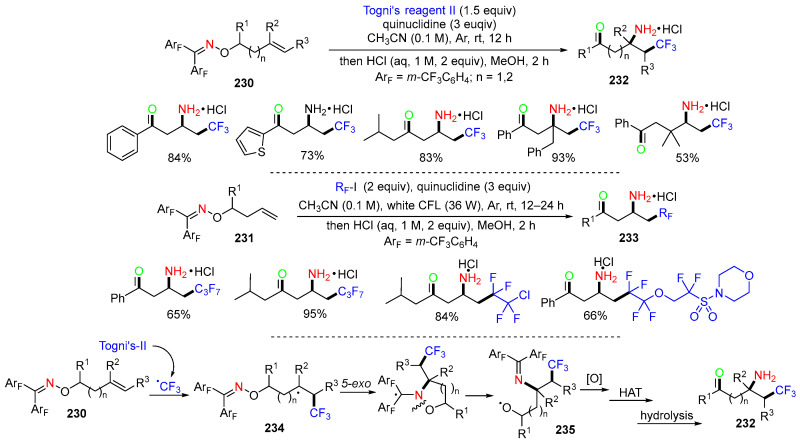
Reaction of alkenyl diphenyl ketoxime ethers.

The Han group, in 2021, introduced a reaction of alkenyl diphenyl ketoximes **230**/**231** for the synthesis of fluoroalkylated aminoketones **232**/**233** (Scheme 61) [94]. The reaction process was initiated with the addition of fluoroalkyl radicals to the C=C bond and involved a 1,4/5-amino shift. In the case of reaction with *Togni’s reagent II*, the CF_3_ radical attacks the C=C double bond of ketoxime ether **230** to form alkyl radicals **234**, followed by a kinetically favorable *5*/*6*-*exo*-*trig* cyclization and N–O bond cleavage to afford oxy radicals **235**. Oxidation of **235**, followed by HAT and hydrolysis, affords the final product **232**. Other than *Togni’s reagent II*, fluoroalkyl iodides could be used for the synthesis of different kinds of fluoroalkyl-substituted products **233**.

**Scheme 62:**
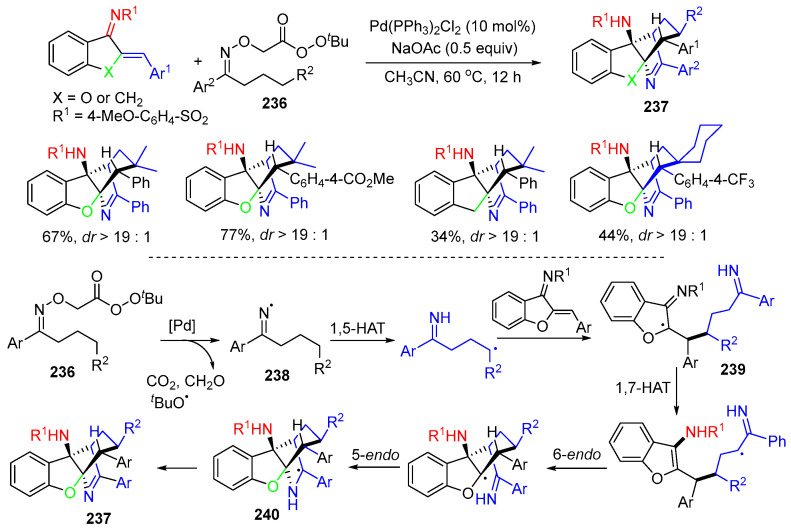
Reaction of oxime-derived peresters with azadienes.

A reaction of oxime-derived peresters **236** with azadienes for diastereoselective synthesis of highly condensed cyclic products **237** was reported by the Wei group in 2022 (Scheme 62) [95]. In the reaction process, the iminyl radicals **238** derived from oxime-derived peresters **236** initiated the cascade radical reactions of 1,5-HAT and addition to azadienes to form **239**, followed by 1,7-HAT, 6-*endo,* and then 5-*endo* cyclizations to give radicals **240**, which then led to the formation of products **237** after SET oxidation.

**Scheme 63:**
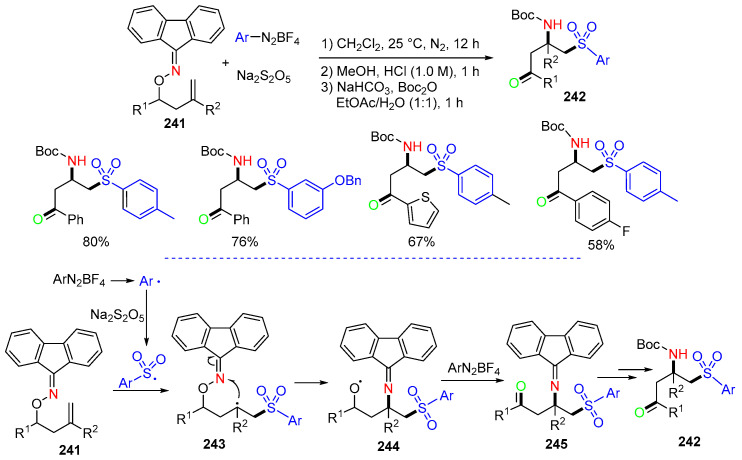
Trifunctionalization of alkenyl oxime ethers.

A reaction of alkenyl oxime ethers **241** with sodium metabisulfite and aryldiazonium tetrafluoroborates for the synthesis of Boc-protected *β*-amino sulfones **242** was reported by the Wu group in 2023 (Scheme 63) [96]. In the alkene aminosulfonylation process, arylsulfonyl radicals, which are generated from the reaction of sodium metabisulfite and aryldiazonium tetrafluoroborates, are added to alkene **241** to form carbon radicals **243**, followed by 1,4-iminyl group migration to give oxy radicals **244**. Oxidative SET with aryldiazonium tetrafluoroborates and protonation afford **245**, which undergo hydrolysis and Boc protection to give Boc-protected *β*-amino sulfone **242**.

**Scheme 64:**
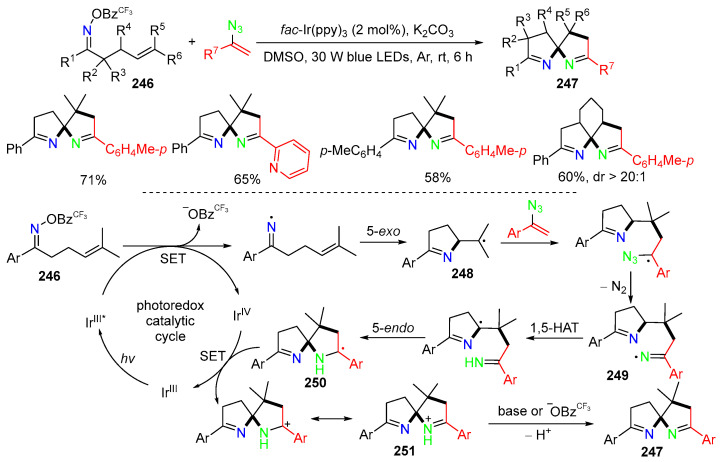
Reaction of oxime estersfor for the synthesis of spirobi[pyrrolines] products.

Other than the oximes and oxime ethers discussed above, oxime esters could be used for the trifunctionalization reaction. A reaction of oxime esters **246** and vinyl azides for the dearomatic synthesis of spiroaminals **247** was developed by the Yi group in 2021 (Scheme 64) [97]. In the reaction process, photoredox-induced iminyl radicals undergo 5-*exo* cyclization to form pyrroline-containing radicals **248**, which are then added to vinyl azides to generate second iminyl radicals **249** after elimination of N_2_. 1,5-HAT of **249**, followed by 5-*endo* cyclization, affords radicals **250** with spiroring scaffold. SET of **250** and proton transfer of **251** produce the spirobi[pyrrolines] products **247**.

**Scheme 65:**
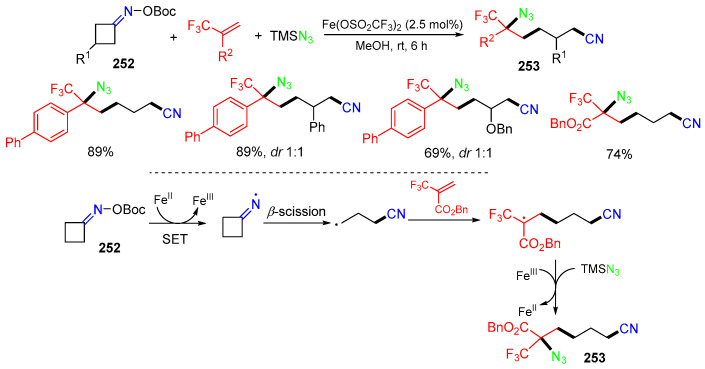
Reaction of cycloketone oxime esters.

The opening of strained rings is a good approach for remote functionalization. In 2023, the Guo group reported a Fe-catalyzed reaction of cycloketone oxime esters **252** with trifluoromethylalkenes and TMSN_3._ The reaction involves a process of cycloketone oxime esters **252** ring opening, radical addition to trifluoromethylalkenes, and azidation with TMSN_3_ to afford products **253** (Scheme 65) [98].

## 8. Summary and Conclusions

Radical trifunctionalization is a relatively new research area. Many of the papers covered in this article were published in the last five years. The effort to promote highly efficient synthesis, product diversity, and sustainable chemistry makes the topic attractive. The development of photoredox catalysis and electrochemical reactions has empowered cascade radical reactions, including di- and trifunctionalization reactions. This article summarizes the different substrates and synthetic strategies developed for radical trifunctionalization reactions. We hope this overview of the field will inspire readers and encourage more chemists to engage in the development of trifunctionalization reactions to make novel scaffolds with potential biological activities and other utilities. We believe that the discovery of new substrate systems and the development of new radical reaction techniques, including photoredox, electrochemical reactions, and transition-metal catalysts, are critical for the future growth of radical trifunctionalization reactions.
